# Ethnic differences in hypertension management, medication use and blood pressure control in UK primary care, 2006–2019: a retrospective cohort study

**DOI:** 10.1016/j.lanepe.2022.100557

**Published:** 2022-12-05

**Authors:** Sophie V. Eastwood, Alun D. Hughes, Laurie Tomlinson, Rohini Mathur, Liam Smeeth, Krishnan Bhaskaran, Nishi Chaturvedi

**Affiliations:** a MRC Unit for Lifelong Health and Aging at UCL, 1-19 Torrington Place, Floor 5, London, WC1E 7HB, UK; b Electronic Health Records Group, London School of Hygiene and Tropical Medicine, 2nd floor, Keppel Street, London, WC1E 7HT, UK

**Keywords:** Hypertension, Antihypertensives, Ethnic differences, Primary care

## Abstract

**Background:**

In the UK, previous work suggests ethnic inequalities in hypertension management. We studied ethnic differences in hypertension management and their contribution to blood pressure (BP) control.

**Methods:**

We conducted a cohort study of antihypertensive-naïve individuals of European, South Asian and African/African Caribbean ethnicity with a new raised BP reading in UK primary care from 2006 to 2019, using the Clinical Practice Research Datalink (CPRD). We studied differences in: BP re-measurement after an initial hypertensive BP, antihypertensive initiation, BP monitoring, antihypertensive intensification, antihypertensive persistence/adherence and BP control one year after antihypertensive initiation. Models adjusted for socio-demographics, BP, comorbidity, healthcare usage and polypharmacy (plus antihypertensive class, BP monitoring, intensification, persistence and adherence for BP control models).

**Findings:**

A total of 731,506 (93.5%), 30,379 (3.9%) and 20,256 (2.6%) people of European, South Asian and African/African Caribbean ethnicity were studied. Hypertension management indicators were similar or more favourable for South Asian than European groups (OR/HR [95% CI] in fully-adjusted models of BP re-measurement: 1.16 [1.09, 1.24]), antihypertensive initiation: 1.49 [1.37, 1.62], BP monitoring: 0.97 [0.94, 1.00] and antihypertensive intensification: 1.10 [1.04, 1.16]). For people of African/African Caribbean ethnicity, BP re-measurement rates were similar to those of European ethnicity (0.98 [0.91, 1.05]), and antihypertensive initiation rates greater (1.48 [1.32, 1.66]), but BP monitoring (0.91 [0.87, 0.95]) and intensification rates lower (0.93 [0.87, 1.00]). Persistence and adherence were lower in South Asian (0.48 [0.45, 0.51] and 0.51 [0.47, 0.56]) and African/African Caribbean (0.38 [0.35, 0.42] and 0.39 [0.36, 0.43]) than European groups. BP control was similar in South Asian and less likely in African/African Caribbean than European groups (0.98 [0.90, 1.06] and 0.81 [0.74, 0.89] in age, gender and BP adjusted models). The latter difference attenuated after adjustment for persistence (0.91 [0.82, 0.99]) or adherence (0.92 [0.83, 1.01]), and was absent for antihypertensive-adherent people (0.99 [0.88, 1.10]).

**Interpretation:**

We demonstrate that antihypertensive initiation does not vary by ethnicity, but subsequent BP control was notably lower among people of African/African Caribbean ethnicity, potentially associated with being less likely to remain on regular treatment. A nationwide strategy to understand and address differences in ongoing management of people on antihypertensives is imperative.

**Funding:**

Diabetes UK.

**Research in context**

**Evidence before this study**

We searched PubMed for observational studies examining associations between ethnicity (search string: ethnic∗ or race or racial or Asian∗ or India∗ or Pakistan∗ or Bangladesh∗ or Black or African or Afro∗) and hypertension/blood pressure (BP)/antihypertensives (hypertens∗ or blood pressure or anti$hypertensive∗) and separately in turn: 1) follow-up of de novo raised blood pressure (detecti∗ or follow∗ or elevated or raised), 2) antihypertensive initiation (initiat∗ or commenc∗ or start∗ or treat∗ or manag∗), 3) blood pressure monitoring whilst on antihypertensive treatment (measure∗ or monitor∗), 4) antihypertensive intensification (intensif∗ or titrat∗ or increase∗ or chang∗ or switch∗ or inertia), 5) antihypertensive persistence (persist∗ or discontinue∗ or stop∗), 6) antihypertensive adherence (adher∗ or compl∗) and 7) blood pressure control on antihypertensives (control or target∗ or achiev∗). A total of 13 relevant studies were found.

Studies from the US have reported ethnic differences in all of the above outcomes, with higher likelihoods of adverse outcomes for people of African or Asian American than white ethnicity. However, their application to UK primary care is hampered by the confounding influence of socio-economic status (highly correlated with ethnicity in the US population) in a fee-paying healthcare system. Furthermore, few studies offered explanations for observed differences.

UK data is limited to ethnic comparisons of BP control, mostly only London-based, which generally report poorer control in people of African/African Caribbean than European ethnicity, and similar or better control for those of South Asian ethnicity. None of these studies sought explanations for observed differences. We are not aware of any UK data comparing hypertension detection, antihypertensive initiation, BP monitoring on treatment, antihypertensive intensification or antihypertensive persistence/adherence by ethnicity.

**Added value of this study**

This is the only UK study that examines ethnic differences in several aspects of hypertension management and antihypertensive use simultaneously. Further, it is one of the largest and most nationally representative UK studies of BP control in people taking antihypertensives, and the only one to seek explanations for differences in control.

We found that guideline-indicated antihypertensive initiation was more likely in people of South Asian or African/African Caribbean than European ethnicity, but that BP monitoring and antihypertensive intensification rates were lower in African/African Caribbean than European groups. However, people of South Asian or African/African Caribbean ethnicity were less likely to remain on treatment over the next year. BP control one year after antihypertensive initiation was less likely for African/African Caribbean than European groups, and similar for people of South Asian ethnicity—the former difference attenuated after accounting for treatment persistence and/or adherence. No ethnic differences were present when the sample was restricted to 131,354 (78%) people in possession of antihypertensive prescriptions covering ≥80% of the first year of treatment.

**Implications of all the available evidence**

Important deficits in some aspects of hypertension management were identified for the African/African Caribbean group, in keeping with US data regarding African American vs white American differences. Nevertheless, differences were not consistent across outcomes; some management outcomes were more favourable in South Asian and African/African Caribbean than European groups, in contrast to US data. Ethnic differences in the likelihood of remaining on regular antihypertensive treatment are reported for the first time in a UK population and are similar in magnitude to those from US data. Poorer BP control in people of African/African Caribbean than European ethnicity, and better control (after adjustment) for those of South Asian ethnicity, correspond with previous UK findings. However, for the first time we offer potential explanations for the lower likelihood of BP control in people of African/African Caribbean than European ethnicity, and show they are absent for people remaining on regular antihypertensive treatment, suggesting a key target for intervention.

## Introduction

Globally, high blood pressure/hypertension affects 25% of the adult population and is the most important modifiable cardiovascular disease (CVD) risk factor.^1^ While, in high-income countries at least, antihypertensives are widely prescribed, evidence suggests that hypertension control may be inequitable, notably by ethnicity.^2^^,^^3^ Systemic racism and structural inequalities are increasingly acknowledged as drivers of ethnic differences in BP control.^2^^,^^4^

Inequalities in clinical management of hypertension may manifest in follow-up of de novo hypertensive blood pressure (BP) readings, antihypertensive initiation, BP monitoring and intensification of antihypertensive treatment. Persistent and adherent antihypertensive use are also key to hypertension control. As far as we are aware, no studies simultaneously compare all these factors by ethnicity, nor examine their contribution to ethnic differences in BP control. Further, UK South Asian and African/African Caribbean populations have far higher rates of diabetes than white European groups^5^ and our previous work suggests ethnic differences in BP control are partly explained by diabetes,^6^ but this requires substantiation in larger datasets.

Using a nationally representative database of UK primary care records, we aimed to determine ethnic differences in hypertension management, antihypertensive use and BP control between people of South Asian or African/African Caribbean versus European ethnicity (the UK's three largest ethnic groups). Our primary objectives were to contrast the following by ethnicity: 1) repeat confirmatory BP measurement after a de novo raised BP, 2) antihypertensive initiation, 3) subsequent antihypertensive management (comprising BP monitoring and treatment intensification), 4) antihypertensive use (comprising persistence and adherence) and 5) BP control. A secondary objective was to study how these parameters varied by diabetes status.

## Method

### Study population

**Fig. 1:**
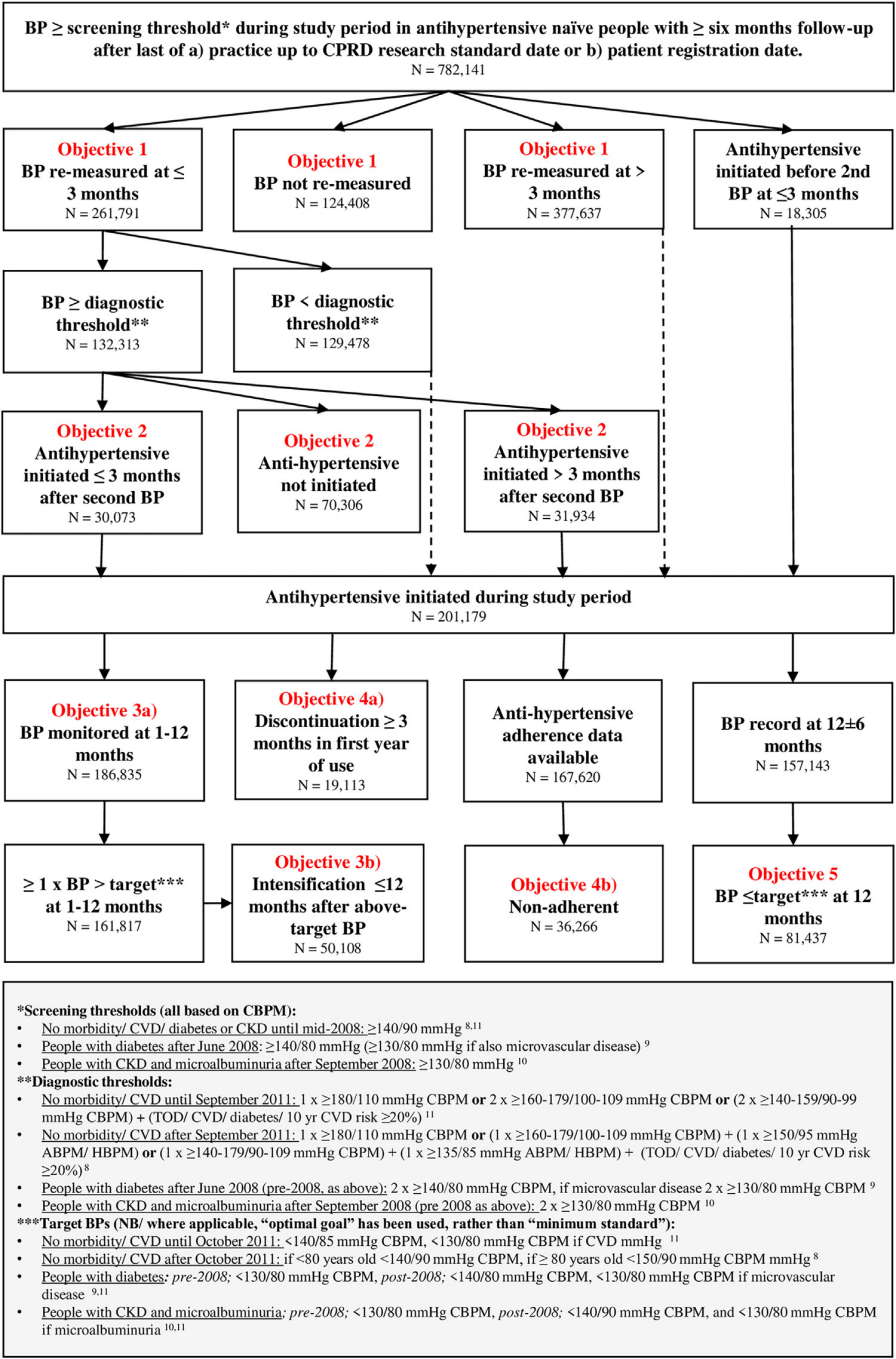
**Flow diagram of hypertension management, antihypertensive use and BP control during the study period (2006–2019), by ethnicity.** Complete case analysis. CPRD = Clinical Practice Research Datalink, CBPM = clinic BP monitoring, ABPM = ambulatory BP monitoring, HBPM = home BP monitoring, CKD = chronic kidney disease, BHS = British Hypertension Society, NICE = National Institute for Health and Care Excellence, TOD = target organ disease, CVD = cardiovascular disease. Dashed lines indicate instances where individuals contribute data to the antihypertensive initiation cohort.

We used data from the Clinical Practice Research Datalink (CPRD) GOLD, a nationally representative database of over 12 million anonymised primary care records from 836 practices.^7^ We selected an adult cohort of people of European, South Asian and African/African Caribbean ethnicity with a first blood pressure (BP) reading exceeding morbidity-dependent thresholds, according to contemporaneous UK clinical guidelines^8^, ^9^, ^10^, ^11^ (see Fig. 1 shaded panel for details) between 1st January 2006 and 30th June 2019. In the UK, all state healthcare is free at the point of delivery; people may have had an incidental BP recording when consulting for another problem, or as part of a routine check (offered 5 yearly to adults over 40 years old). Initial BPs were over 6 months after the latest of i) the patient's current registration date or ii) the practice's up to CPRD standard date (Figure S1) to ensure incident cases. We restricted the sample to antihypertensive naïve individuals with complete data (Figure S1 for cohort derivation, Table S1 for definition of prevalent antihypertensive use).

### Exposure

The main exposure was self-reported European, South Asian, or African/African Caribbean ethnicity, designated by Read codes (further detail in Table S1). We have previously demonstrated that CPRD is representative of census-derived ethnicity proportions in the UK population.^12^ Ethnic sub-groups were also derived: British, Irish, other white, Indian, Pakistani, Bangladeshi, other South Asian, Caribbean, African, and other Black. People without an ethnicity Read code or ethnicities other than those above, were excluded (Figure S1).

### Outcomes

#### Repeat confirmatory BP measurement

Repeat BP measurement within three months of a de novo elevated BP^8^^,^^11^ was compared by ethnicity (N = 782,141, Fig. 1). People initiating antihypertensives prior to a second recorded BP were excluded from this analysis (N = 18,305, Fig. 1).

#### Antihypertensive initiation

Antihypertensive initiation within three months of the second BP reading^8^^,^^11^ was contrasted by ethnicity for 132,313 people in whom contemporaneous guidelines would have indicated antihypertensive use. Again, those initiating antihypertensives prior to a second BP were excluded to ensure parity of study entry dates. Individuals with a second BP measurement exceeding the diagnostic thresholds recommended by contemporaneous UK guidelines^8^, ^9^, ^10^, ^11^ (see shaded panel in Fig. 1) were included in the denominator of the antihypertensive initiation analyses if the following were present (according to time-updated covariates derived at the date of the second BP): a) stage 1 hypertension plus 10 year CVD risk ≥20% or target organ disease (CVD, CKD, left ventricular hypertrophy, hypertensive retinopathy, proteinuria or haematuria), b) stage 2 hypertension or c) BP exceeding disease-specific thresholds for people with diabetes or CKD. A sensitivity analysis examined antihypertensive initiation within 6 months of the second BP.

From September 2011, UK guidelines advocated hypertension ascertainment (after a first raised clinic BP reading) via ambulatory (ABPM) or home (HBPM) BP monitoring.^8^ Practices usually enter mean ABPM values into BP templates, thus ABPM Read codes are rarely recorded; only 1687/132,313 (1.3%) of second BP readings had an ABPM code. We assumed all readings after September 2011 were from ABPM or HBPM, supported by data from a 2017 survey of English general practitioners which reported 74% ABPM/HBPM uptake,^13^ and thus employed (lower) ABPM thresholds for antihypertensive initiation (see shaded panel in Fig. 1). This assumption was tested in sensitivity analyses using i) second BPs coded as ABPM or ii) clinic BP diagnostic thresholds.

Initiation of angiotensin-converting enzyme inhibitors/angiotensin receptor blocking drugs, calcium channel blockers, thiazide diuretics or combinations of these was studied, commensurate with recommendations for first to third line use during the study period.^14^

#### Subsequent antihypertensive management

The number of BP measurements in the first year after antihypertensive initiation was compared by ethnicity for the 201,179 individuals who initiated antihypertensives during the study period (Fig. 1). Following this, time to antihypertensive intensification in the first year of use was compared for 161,817 individuals (80% of those initiating antihypertensives, Fig. 1) with an above-target BP. Intensification was defined as addition of or switching to a different antihypertensive class (not dose change).^8^^,^^11^

#### Antihypertensive use

Persistence (defined as no gaps in antihypertensive prescriptions exceeding 90 days during the first year of treatment) was studied for the 201,179 people commencing antihypertensives (Fig. 1). Additionally, adherence was assessed using the proportion of days covered^15^ (PDC) during the first year of treatment for the 167,620 people with available data (Fig. 1); PDC exceeding 80% was deemed adherent. Sensitivity analyses used a six-month prescription gap cut-point for persistence and the medication possession ratio^15^ (MPR) to assess use (Table S1 for PDC/MPR derivation). Sub-group analyses compared use measures by age group, gender, deprivation and antihypertensive class.

#### Blood pressure control

We evaluated ethnic differences in BP control one year after antihypertensive initiation in the 157,143 people with a BP at 12 ± 6 months, using the BP reading closest to the date of antihypertensive initiation plus 365 days (Fig. 1). Control was defined as a BP reading at or below the morbidity-dependent treatment target (shaded panel in Fig. 1). A sub-group analysis compared associations between ethnicity and BP control by crossed categories of antihypertensive persistence and adherence (i.e., non-persistent + non adherent, non-persistent + adherent, persistent + non-adherent and persistent + adherent).

### Covariates

The following covariates were selected to explore what factors might explain ethnic differences in outcomes (see direct acyclic graph, Figure S2): sociodemographic factors (age, gender, deprivation), CVD risk factors (smoking, systolic [SBP] and diastolic [DBP] blood pressure, BMI, statin use), comorbidity (diabetes, chronic kidney disease, cardiovascular disease, serious mental illness, cancer and asthma/chronic obstructive pulmonary disease), healthcare usage (consultation rate), polypharmacy (number of medications) and antihypertensive class; further details in Table S1. The index date for objectives two to five was the date of antihypertensive initiation, thus time-updated covariates were derived at this point.

### Statistical analysis

All analyses were pre-specified in a scientific protocol and implemented using Stata, version 17.

The study sample was drawn from those with available ethnicity codes; the only variables in the sample with missing data were smoking status and BMI, which relied solely on physicians’ recording, and thus were not complete for all individuals. We used complete case analysis to handle missing data, see Table S1 for details, as we believe its central assumption (conditional independence between missingness and outcome) is more plausible than the “missing at random” assumption needed for multiple imputation. BMI and smoking status in primary care data are unlikely to be missing at random (i.e., higher BMIs/current smoking status are more likely to be recorded).

Additionally, for eligible individuals in the entire CPRD (i.e., adults with a first BP reading exceeding morbidity-dependent thresholds between 2006 and 2019), ethnicity data were missing for approximately 48% (Figure S1). To examine the influence of missing ethnicity, we performed a sensitivity analysis comparing ethnic differences in all outcomes by practices with above and below median (44%) ethnicity recording rates.

Baseline characteristics were compared by ethnicity and between the complete case analysis sample and the samples for which i) BMI/smoking data, ii) ethnicity data, and iii) adherence data were missing.

BP re-measurement within three months of a de novo raised BP, antihypertensive initiation within three months of second BP, antihypertensive persistence, antihypertensive adherence and BP control at one year were assessed using logistic models. BP monitoring was evaluated using negative binomial models (data were too over-dispersed for Poisson models). Antihypertensive intensification was studied using Cox models; the entry date was the first above-target BP after antihypertensive initiation in the first year of treatment and the exit/censoring date was the first of: the outcome (antihypertensive intensification), study end date (30th June 2019), death, transfer to a different practice or practice last collection date. Proportional hazards assumptions were verified by inspection of Kaplan–Meier cumulative survival plots by ethnic group (Figure S3) and performing formal tests of Schoenfeld residuals.

Hypertension management behaviour for clinicians within a primary care practice may be correlated, and drive outcomes such as prescribing and BP control independently of patient factors such as ethnicity. To address this potential clustering of behaviour, we used cluster-robust standard errors, which widen confidence intervals to reflect a greater degree of uncertainty for the main (i.e., ethnicity) estimate, in view of the potential influence of the clustering variable (practice). We elected not to use random effects models as investigating the extent of intra-practice correlation was beyond the scope of this study.

All models were adjusted for i) age and gender, ii) age, gender and SBP and iii) age, gender, SBP, deprivation, CVD risk factors, comorbidity, healthcare usage and polypharmacy. Antihypertensive persistence and adherence models were additionally adjusted for antihypertensive class in model iii). To explore possible explanations, BP control models were adjusted for the following additional factors separately in turn added to model ii): deprivation, diabetes, antihypertensive class, BP monitoring frequency, intensification and persistence; then a final model combined them all. BP control was also assessed in the nested cohort with available adherence data (N = 167,620), both adjusting for adherence and stratifying on adherence status.

Pre-specified secondary sub-group analyses were performed to study whether ethnic differences in all outcomes varied by diabetes status, ethnic sub-group or calendar time period (categorised as: i) 1st January 2006 to 31st August 2011 [when UK guidelines changed to incorporate ambulatory BP monitoring], ii) 1st September 2011 to 31st January 2015 and iii) 1st February 2015 to 30th June 2019; outcomes were examined for the whole sample by calendar time period in addition to ethnic differences in outcomes). We also performed sensitivity analyses examining all outcomes using BP screening, diagnostic and treatment thresholds in operation at the study outset.

### Ethical approval

Ethical approval for this study was obtained from the Independent Scientific Advisory Committee (protocol 19_045) and the London School of Hygiene & Tropical Medicine (project ID 14222).

### Role of funding source

The funder had no role in study design, collection/analysis/interpretation of data, writing the report or the decision to submit the manuscript for publication.

## Results

Of the 5,566,120 individuals in the CPRD with recorded European, South Asian or African/African Caribbean ethnicity, 882,859 were adults with at least six months prior registration and an above-threshold BP (at which point they were antihypertensive-naïve) during the study period (Figure S1). After exclusions due to incomplete data (n = 100,718, 11%), 782,141 people remained. Comparison of baseline characteristics of the complete case versus missing data samples revealed minor differences, and missingness was similar by ethnicity (Table S2a). People with missing ethnicity (n = 747,184) were excluded from the study; their characteristics are compared in Table S2b. The sensitivity analysis of all outcomes by practice ethnicity recording rate revealed no major differences between practices with high versus low recording rates (Table S2c). Comparison of baseline characteristics by availability of adherence data showed higher levels of missingness for people of South Asian and African/African Caribbean, compared to those of European, ethnicity (N = 1868 [24%] and N = 1502 [27%] versus N = 30,189 [16%] respectively, p < 0.001), though other characteristics were mostly similar (Table S2d).

People of South Asian and African/African Caribbean ethnicity were younger than those of European ethnicity, and deprivation levels greatest in the African/African Caribbean group (Table 1). SBP was lower in people of South Asian than European ethnicity, and similar in those of African/African Caribbean ethnicity. The study sample was selected using morbidity-dependent BP thresholds, which are lower for people with diabetes. Diabetes was approximately three times more common in the South Asian than European groups, therefore BPs for people of South Asian ethnicity in the study sample were lower than would be expected at a population level. Diabetes was also twice as prevalent in people of African/African Caribbean than European ethnicity. Patterns were identical for time-updated covariates in those commencing antihypertensives (where antihypertensive initiation was the study entry date; thus characteristics were assessed at that timepoint); the expected preponderance of CCB use in African/African Caribbean groups was observed^8^ (Table S3).

**Table 1:** Baseline characteristics of antihypertensive-naïve people with at least one BP exceeding screening threshold during the study period (2006–2019), by ethnicity.

	European ethnicity	South Asian ethnicity	African/African Caribbean ethnicity
N (%)	731,506 (93.5)	30,379 (3.9)	20,256 (2.6)
Age, yrs	52 (15)	46 (13)	44 (12)
Female gender	387,619 (56)	14,553 (49)	11,000 (54)
Index of multiple deprivation quintile:			
1 (least deprived)	118,819 (16)	3203 (10)	964 (5)
2	131,896 (18)	3749 (12)	1897 (9)
3	140,657 (19)	6305 (21)	3235 (16)
4	151,558 (21)	8488 (28)	5997 (29)
5 (most deprived)	188,576 (26)	8634 (28)	8163 (39)
**Cardiovascular disease risk factors**			
Smoking:			
Never	320,299 (45)	20,286 (67)	13,119 (65)
Ex	256,214 (34)	6363 (21)	4447 (23)
Current	154,993 (21)	3730 (12)	2690 (12)
Systolic blood pressure (1st reading):			
Mean ± SD, mmHg	146 (14)	143 (14)	146 (14)
<140 mmHg	132,683 (20)	10,438 (31)	5215 (24)
140–149 mmHg	371,290 (51)	13,336 (45)	9562 (46)
150–159 mmHg	119,359 (15)	3763 (13)	3041 (16)
160+ mmHg	108,174 (14)	2842 (11)	2438 (14)
Diastolic blood pressure (1st reading):			
Mean ± SD, mmHg	87 (10)	87 (9)	88 (10)
80–89 mmHg	399,867 (56)	15,167 (55)	9637 (54)
90–99 mmHg	259,220 (35)	12,344 (37)	8103 (35)
100–110 mmHg	59,248 (7)	2312 (7)	2005 (8)
110+ mmHg	13,171 (2)	556 (2)	511 (2)
BMI, kg/m^2^	28 (6)	27 (5)	29 (6)
Statin use	65,802 (8)	4316 (15)	1275 (10)
**Comorbidity**			
Diabetes	46,140 (7)	5673 (19)	2186 (14)
CKD	43,234 (7)	2374 (9)	205 (3)
CVD	20,357 (4)	476 (3)	198 (3)
Serious mental illness	23,553 (3)	750 (2)	786 (4)
Cancer	32,163 (5)	483 (2)	347 (4)
Asthma/COPD	90,354 (13)	2897 (11)	1556 (9)
**Healthcare usage**			
No. consultations in previous year:			
Median (IQR)	3 (1, 7)	4 (2, 8)	4 (1, 7)
0–1 consultations	216,766 (27)	7543 (23)	5681 (25)
2–5 consultations	262,721 (36)	10,542 (34)	7540 (36)
5+ consultations	252,019 (37)	12,294 (43)	7035 (39)
**Polypharmacy**			
No. medications in previous year:			
Median (IQR)	1 (1, 2)	1 (1, 2)	1 (0, 2)
0 medications	160,445 (20)	6640 (20)	5432 (24)
1–3 medications	275,361 (37)	9951 (30)	7436 (33)
4+ medications	295,700 (43)	13,788 (50)	7388 (43)

Data are n (age-standardised %) or age-adjusted mean (SD) unless otherwise stated, complete case analysis.

CKD = chronic kidney disease, COPD = chronic obstructive pulmonary disease.

### Repeat confirmatory BP measurement

**Fig. 2:**
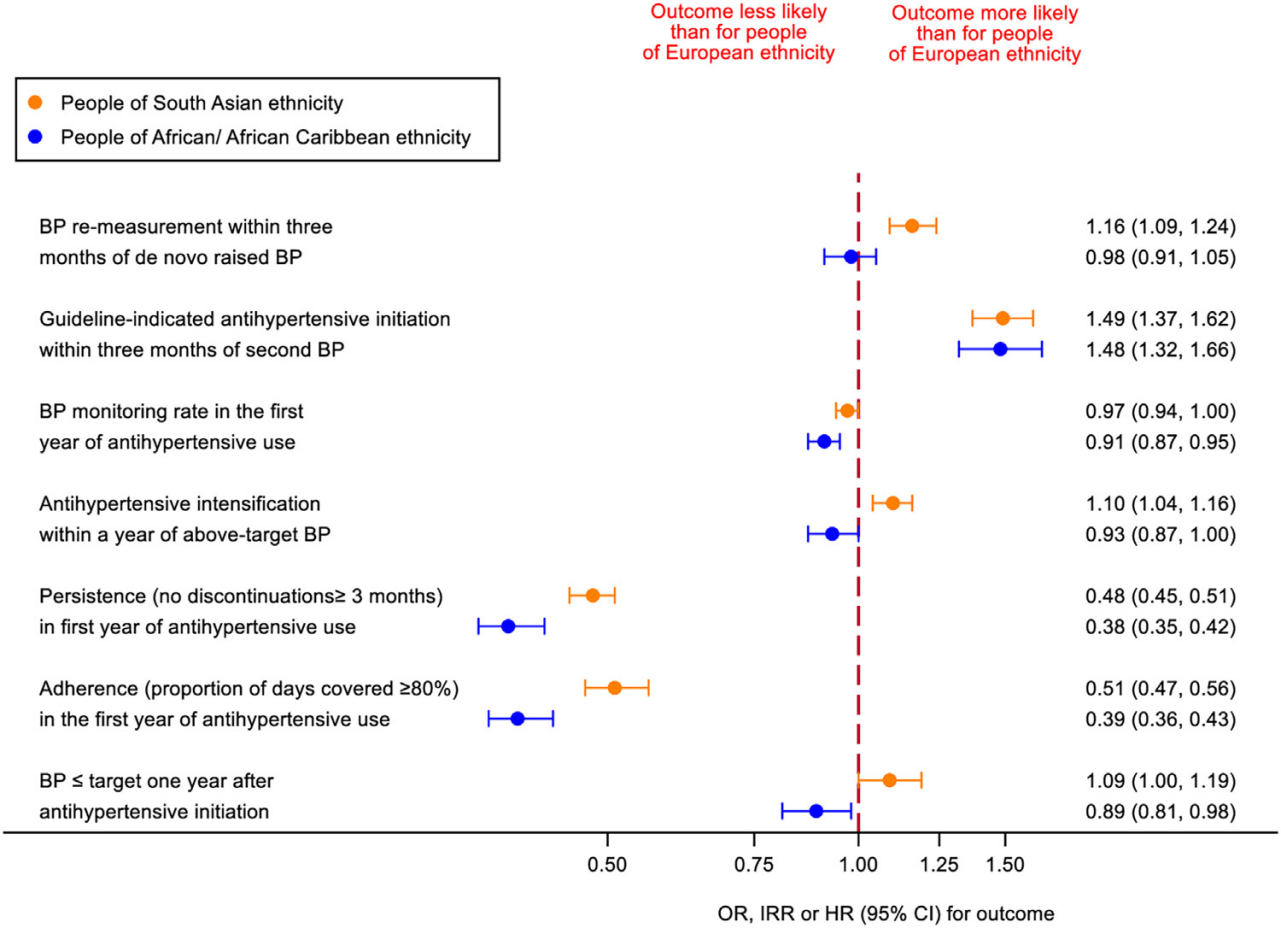
**Ethnic differences in hypertension management, antihypertensive usage and BP control at one year.** Data are OR, IRR (BP monitoring rate), or HR (intensification) (95% CI), complete case analysis. Models (fully) adjusted for: age, gender, SBP, deprivation, cardiovascular disease risk factors, comorbidity, healthcare usage and polypharmacy. Persistence and adherence models also adjusted for antihypertensive class, BP control model also adjusted for antihypertensive class, monitoring frequency, intensification and persistence. Intensification data for nested cohort of individuals with a BP exceeding treatment target ≥30 days after antihypertensive initiation (N = 161,817). Adherence data for nested cohort of individuals with usable adherence measures (N = 167,620). BP control data for nested cohort with follow-up BP available at 12 ± 6 months after antihypertensive initiation (N = 157,143).

BP re-measurement within three months of a de novo raised BP was more likely for people of South Asian than European ethnicity (age and gender-adjusted OR [95% CI]: 1.07 [1.01, 1.12]); the difference increased in fully-adjusted models (1.16 [1.09, 1.24], Fig. 2, Table S4), mostly driven by adjustment for SBP (Table S4)—lower in the former group. BP re-measurement was similar people of African/African Caribbean and European ethnicity in both age and gender- (1.01 [0.95, 1.07], Table S4) and fully-adjusted models (0.98 [0.91, 1.05], Fig. 2, Table S4).

NB/ those initiating an antihypertensive prior to the second BP reading were excluded at this stage, but we note this was more likely for people of both South Asian (N = 785, 2.6%) and African/African Caribbean (N = 695, 3.4%) than European (N = 16,825, 2.3%) ethnicity (age and sex-adjusted ethnicity OR [95% CI]: 1.24 [1.16, 1.33] and 1.76 [1.63, 1.89] respectively).

### Antihypertensive initiation

In age and gender adjusted models, antihypertensive initiation was similarly likely for people of European and South Asian ethnicity (0.95 [0.88, 1.03]) and more likely for those of African/African Caribbean ethnicity (1.33 [1.21, 1.47]) (Table S4). Again, after adjustment for SBP, initiation rates exceeded those of European groups for both South Asian and African/African Caribbean groups and did not alter greatly with full adjustment (1.49 [1.37, 1.62] and 1.48 [1.32, 1.66] respectively Fig. 2, Table S4).

### Subsequent antihypertensive management

BP monitoring rates in the first year of antihypertensive treatment were lower for South Asian and African/African Caribbean than European groups (age and gender adjusted IRR [95% CI] 0.94 [0.91, 0.97] and 0.93 [0.89, 0.97], Table S4). The ethnic difference attenuated with adjustment for SBP and in the fully adjusted model for people of South Asian ethnicity (0.97 [0.94, 1.00], Fig. 2, Table S4), but remained for those of African/African Caribbean ethnicity (0.91 [0.87, 0.95], Fig. 2, Table S4).

Time to antihypertensive intensification was longer and antihypertensive intensification rates were lower for people of South Asian or African/African Caribbean than European ethnicity (median [IQR] days to intensification: 87 [36,179], 84 [32,184] and 77 [30,174] respectively, rates per 1000 person years at risk [95% CI]: 0.41 [0.39, 0.43], 0.40 [0.38, 0.43] and 0.44 [0.43, 0.44] respectively, age and gender adjusted HR [95% CI]: 0.96 [0.91, 1.01] and 0.93 [0.87, 0.99], Table S4). After full adjustment, intensification was more likely for people of South Asian than European ethnicity (1.10 [1.04, 1.16]), but remained less likely for the African/African Caribbean group (0.93 [0.87, 1.00], Fig. 2, Table S4).

### Antihypertensive use

People of South Asian or African/African Caribbean ethnicity were less likely to remain on regular antihypertensive treatment than those of European ethnicity, regardless of adjustment (fully-adjusted OR [95% CI] for persistence: 0.48 [0.45, 0.51] and 0.38 [0.35, 0.42], and for adherence: 0.51 [0.47, 0.56] and 0.39 [0.36, 0.43] respectively, Fig. 2, Table S4).

### Blood pressure control

At 12 ± 6 months after antihypertensive initiation, 76,495 (52%), 3087 (52%) and 1855 (46%) people of European, South Asian and African/African Caribbean ethnicity respectively had controlled BPs (Table S4).

**Fig. 3:**
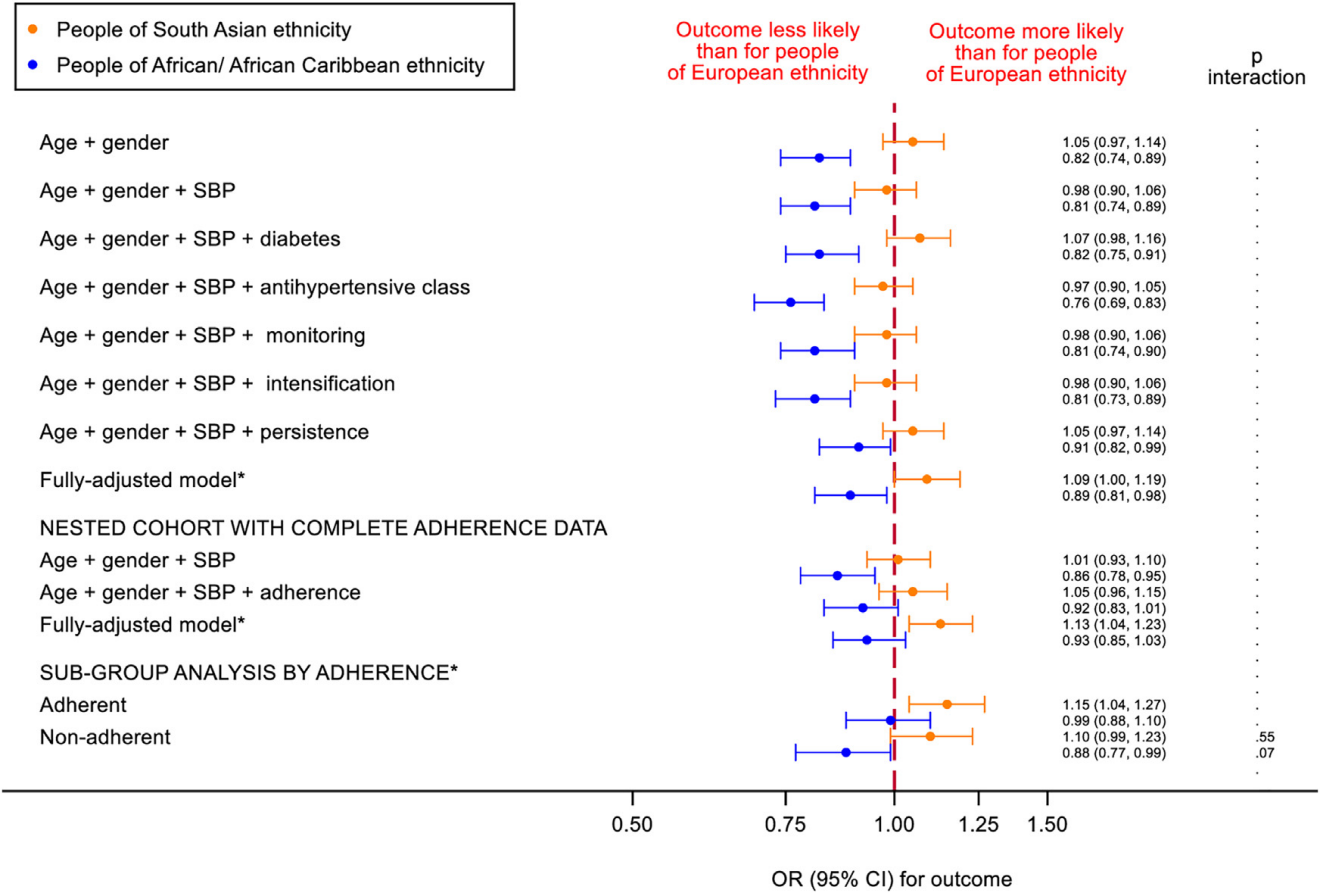
**Associations between ethnicity and BP control one year after antihypertensive initiation.** Data are OR (95% CI), complete case analysis of nested cohort with follow-up BP available at 12 ± 6 months after antihypertensive initiation (N = 157,143). ^∗^Fully adjusted model factors: age, gender, SBP, deprivation, cardiovascular disease risk factors, comorbidity, healthcare usage, polypharmacy, antihypertensive class, monitoring, intensification, persistence (the latter not for persistence sub-group analysis). Adherence data for nested cohort of individuals with available follow-up BPs and usable adherence measures (N = 140,549).

In an age and gender-adjusted model, BP control was similarly likely for people of South Asian and European ethnicity (OR [95% CI]: 1.05 [0.97, 1.14]). The difference was unaffected by further adjustment for SBP, deprivation, diabetes, antihypertensive class, BP monitoring frequency, intensification or persistence (Fig. 3, Table S4), but control appeared more likely in the South Asian than European groups in the fully-adjusted model (1.09 [1.00, 1.19]), Similar patterns were seen in the nested cohort with adherence data, with little evidence for differences in control in the adherent versus non-adherent sub-groups (Fig. 3, Table S4).

The likelihood of BP control was lower for people of African/African Caribbean than European ethnicity in an age and gender-adjusted model (0.82 [0.74, 0.89]); this was unaffected by further adjustment for SBP, deprivation, diabetes, antihypertensive class, BP monitoring frequency or intensification, though attenuated by adjustment for persistence (0.91 [0.82, 0.99], Fig. 3, Table S4); the estimate remained similar in the fully-adjusted model. In the nested cohort with adherence data, BP control was also less likely for people of African/African Caribbean than European ethnicity (0.86 [0.79, 0.96] in an age and gender-adjusted model); this was attenuated by further adjustment for adherence and in the full model (0.93 [0.85, 1.03]). When stratified by adherence, no ethnic difference was present for those remaining on regular treatment (0.99 [0.88, 1.10], fully-adjusted), though poorer control remained for those who did not (0.88 [0.77, 0.99], interaction p = 0.07), see Fig. 3, Table S4. The results of the sub-group analysis by crossed categories of antihypertensive persistence and adherence are shown in Table S5.

### Interaction with diabetes

Sub-group analyses showed that, for people with diabetes of South Asian ethnicity, BP re-measurement was more likely than for European groups, and to a greater extent than for people without diabetes (ethnicity × diabetes interaction p = 0.02), but that other outcomes were similar by diabetes status (Figure S4a, Table S6).

People with diabetes of African/African Caribbean ethnicity were more likely to have BP re-measured than their European counterparts, whereas no ethnic difference was present for people without diabetes (interaction p = 0.05, Figure S4b, Table S6). People with diabetes of African/African Caribbean ethnicity were also more likely than those of European ethnicity to have antihypertensive treatment intensified (1.22 [1.01, 1.40]), in contrast to those without diabetes where this outcome was less likely (0.89 [0.83, 0.96], interaction p < 0.001). Ethnic differences in antihypertensive persistence were also less marked in people of African/African Caribbean versus European ethnicity with diabetes than those without (interaction p = 0.04), though similar for all other outcomes.

### Other sensitivity and sub-group analyses

Analyses of all outcomes stratified by calendar time period are shown in Table S7 and Figures S5 and S6; these showed some diminution of ethnic differences in antihypertensive adherence over time, but broadly patterns in other outcomes were similar over time. No major deviations from the main analyses were found in sensitivity analyses of hypertensive initiation (Table S8) and antihypertensive use (Table S9). The sensitivity analyses examining all outcomes using BP screening, diagnostic and treatment thresholds in operation at the study outset found no deviations from the main results (Table S10). Sub-group analyses of antihypertensive use showed lower levels of persistence and adherence in younger age groups, men and those initiating thiazides or calcium channel blockers (persistence only), regardless of ethnicity (Table S11). Generally, only minor differences in all outcomes were present between ethnic sub-groups when compared to the corresponding main ethnic group (Table S12).

## Discussion

In the UK's most comprehensive comparison of hypertension management by ethnicity, we report the following novel findings: 1) guideline-indicated antihypertensive initiation was 50% more likely for people of South Asian or African/African Caribbean than European ethnicity, however, 2) BP monitoring and antihypertensive intensification rates were 10% lower for people of African/African Caribbean than European ethnicity, 3) South Asian and African/African Caribbean groups were less likely than the European group to continue on regular antihypertensive treatment during the first year and 4) BP control one year after antihypertensive initiation was less likely for people of African/African Caribbean than European ethnicity—though equivalent in the population with regular continual antihypertensive use. For people with diabetes, ethnic differences were generally minimal or more likely to favour the South Asian and African/African Caribbean groups.

No recent UK studies report follow-up of de novo elevated BP by ethnicity, but older studies indicate increased hypertension detection in people of African/African Caribbean^16^ but not South Asian ethnicity,^17^ in contrast to our findings. Differences may be related to study populations, vintage or size. However, our finding of higher rates of timely antihypertensive initiation for people of South Asian or African/African Caribbean than European ethnicity is corroborated by previous UK cross-sectional studies demonstrating higher proportions of treated hypertension in the former ethnic groups.^6^^,^^16^ Furthermore, a systematic review of studies from Europe showed similar rates of treatment for South Asian and European groups, and higher rates in African groups, partly supporting our findings.^18^ Explanations are uncertain; a possibility is physician perception of heightened CVD risk in these groups, following the propagation of guidelines and risk scores emphasising this.^8^

UK data on ethnic differences in hypertension monitoring is limited to one study comparing presence of an annual BP reading by ethnicity.^19^ The authors found no ethnic differences, commensurate with our findings for South Asian groups, but we found BP monitoring rates were around 10% lower for people of African/African Caribbean than European ethnicity. Some studies describe lower healthcare usage in African/African Caribbean than other ethnic groups,^20^ but consultation rates did not vary by ethnicity in this study. However, measurement of BP monitoring may have been confounded by home monitoring, which may vary by ethnicity. Furthermore, low BP monitoring rates may indicate poor healthcare engagement or rapid hypertension control rather than sub-optimal healthcare, and high BP monitoring rates may reflect treatment-resistant hypertension rather than better healthcare, therefore these results should be interpreted cautiously.

We are not aware of any UK research comparing antihypertensive intensification by ethnicity, but a recent US study described higher antihypertensive intensification rates in “Asian” than white Americans, and lower rates for African Americans, in keeping with our findings. Intensification may have been less likely for people of African/African Caribbean than European ethnicity in this study due to the observed lower BP monitoring rates post antihypertensive initiation in the former, though importantly it was absent for people with diabetes, in whom intensification was more likely, possibly due to more frequent reviews.

We report ethnic differences in apparent antihypertensive use. Persistence/adherence measures from prescribing records only approximate actual levels of medication taking; our data preclude investigation of medication acquisition from pharmacies or medication consumption,^15^ and we note that electronic prescribing record data has been shown to overestimate medication taking (as quantified by serum assays) by up to 30%.^21^ Further, our adherence results should be interpreted with caution as there were higher levels of missing adherence data for people of South Asian and African/African Caribbean, compared to those of European, ethnicity (though persistence data was complete in all). Nevertheless, in this study both South Asian and African/African Caribbean groups were less likely than European groups to remain on regular treatment during the first year of use. UK data on this are scant,^22^ but numerous North American studies report sub-optimal antihypertensive adherence in South Asian and African American populations.^23^^,^^24^ In our study, the South Asian and African/African Caribbean groups were far younger than the European group, and CCB initiation was much more likely for the African/African Caribbean than other groups; we demonstrated both factors were associated with less optimal antihypertensive use (though adjustment for them could not fully explain ethnic differences in control). Another potential contributory factor that we were unable to explore is ethnic variation in antihypertensive tolerability. However, our data also showed some diminution of ethnic differences in persistence and adherence over time, although we note differences of at least 40% still remained in the latter time periods. US research highlights structural racism as a driver of ethnic differences in medication adherence, manifest in sub-optimal follow-up, poor clinician/patient communication and resultant mistrust of healthcare providers.^2^^,^^25^

UK and European studies have described poorer BP control in people of African/African Caribbean compared to European ethnicity,^3^^,^^16^^,^^18^^,^^19^^,^^26^, ^27^, ^28^ and equivalent or better BP control for those of South Asian ethnicity.^3^^,^^18^^,^^19^^,^^26^, ^27^, ^28^ Our findings corroborate previous work, but we provide an explanation for the lower likelihood of BP control in African/African Caribbean compared with European groups; the difference in medication use by ethnicity. No ethnic difference was present when the sample was restricted to individuals with continual regular antihypertensive use. However, our finding of equivalent BP control for people of South Asian and European ethnicity was puzzling, given the lower adherence in the former. This may have been driven by better BP control in the later (2015–2019) time period. Other explanations may include over-diagnosis and/or prescribing for South Asian, compared to other, groups (possibly driven by white-coat hypertension), their lower baseline BP being easier to control or a requirement for lower doses of antihypertensives.

Of note, we found between 46% and 52% of individuals had achieved BP control one year after antihypertensive initiation, depending on ethnicity. These proportions exceed those reported from a national cohort (38.1% of those taking antihypertensives were controlled in the UK Biobank study)^28^ and survey (the 2015 Health Survey for England reported 35% were controlled)^29^ data. Both studies were cross-sectional (with voluntary participation which may have introduced selection bias), and therefore incorporation of data from people who have taken antihypertensives for multiple years likely explains the lower rates of control when compared with our study.

Key strengths of this study include a large, nationally representative population, with prospective electronic prescribing data. We studied several outcomes to identify management inequity at various stages and used these as potential explanatory variables for ethnic differences in BP control; no previous UK study has sought explanations for BP control differentials.^3^^,^^16^^,^^19^^,^^26^, ^27^, ^28^ Most UK primary care prescriptions are electronically recorded, so ascertainment of antihypertensive prescriptions was likely near complete. However, as with all studies of electronic health records, some selection bias is likely, as more data on covariates such as BMI and smoking are recorded for regular attenders, who may also have more health problems^30^; nevertheless, our comparison of baseline characteristics for those with versus without missing covariate data did not reveal any major differences. Additionally, this study used only data from people with a recorded ethnicity of interest; we believe the complete case assumption (conditional independence between missingness and outcome^31^) is more plausible than the missing at random assumption needed for multiple imputation, since missingness of ethnicity in primary care data is likely to be associated with ethnicity itself. Reassuringly, our comparison of the main results by practice ethnicity recording rate revealed no major differences. People of South Asian and African/African Caribbean ethnicity are the UK's second and third largest ethnic groups; a further limitation was the inability to study ethnic differences for other ethnic groups due to small sample sizes.

In summary, more people of UK South Asian or African/African Caribbean ethnicity started guideline-indicated antihypertensive treatment than their European counterparts, but fewer people of African/African Caribbean than European ethnicity received appropriate antihypertensive monitoring and intensification. People of South Asian or African/African Caribbean ethnicity appeared less likely to continue regular antihypertensive treatment in the first year of use than European groups. BP control was poorer for people of African/African Caribbean than European ethnicity; this was explained by differences in antihypertensive use. The UK national healthcare system is universal and free at the point of delivery, thus ameliorating some barriers to care that may drive ethnicity-related health inequalities elsewhere. Nonetheless, other facets of structural racism may have contributed to the observed inequities at various stages of care. Further, it is crucial that ethnicity is recorded when patients are registered with primary care practices, to ensure that ethnic inequalities in disease management can be adequately identified and tackled. Ethnic differences in BP control are likely to correlate with unacceptable downstream excess CVD risks, therefore a nationwide strategy is imperative for addressing inequalities in antihypertensive management and use with targeted interventions.

## Contributors

All authors were involved in the study design. SVE did the literature search, statistical analysis and wrote the first draft. All authors were involved in data interpretation, contributed to further drafts and approved the final manuscript. KB has directly accessed and verified the underlying data reported in the manuscript.

## Data sharing statement

Data were obtained from the CPRD (www.cprd.com). CPRD is a research service that provides primary care and linked data for public health research. CPRD data governance and our own licence to use CPRD data do not allow us to distribute or make available patient data directly to other parties. Researchers can apply for data access with CPRD and must have their study protocol approved by the Independent Scientific Advisory Committee for Medicines and Healthcare products Regulatory Agency database research.

## Declaration of interests

SVE is funded by a 10.13039/501100000361Diabetes UK Sir George Alberti research training fellowship (grant number: 17/0005588). RM reports personal fees from 10.13039/100002429Amgen, outside the submitted work. NC has received personal fees from 10.13039/100004325AstraZeneca outside of the submitted work. All other authors have no competing interests to declare.

## Appendix A Supplementary data

Supplementary Tables and Figures

STROBE-checklist
