# Supplementary material for: Ethnic differences in hypertension management, medication use and blood pressure control in UK primary care, 2006–2019: a retrospective cohort study
Source: Lancet Reg Health Eur. 2022 Dec 5;25:100557. doi: 10.1016/j.lanepe.2022.100557 (PMC9929586; doi:10.1016/j.lanepe.2022.100557)
Supplement: Supplementary Tables and Figures [file mmc1.docx]

**Supplementary material**

**Table S1. Supplementary methods.**

| **STUDY POPULATION** |
| --- |
| **Definition of prevalent antihypertensive use** |
| Any prescription of medication/s from the following classes prior to the first raised BP reading: angiotensin-converting enzyme inhibitors/ angiotensin receptor blocking drugs, beta-blockers, calcium channel blockers, thiazide diuretics and combinations of these, commensurate with contemporary NICE guidelines at the start of the study period. ^1^ See table S1 for numbers of people excluded due to prevalent antihypertensive use.  Note this contrasts with the definition of incident antihypertensive use, which comprised initiation of medication from the above classes excluding beta-blockers, commensurate with the retirement of this class from first to third line use in primary care early in the study period. ^2^ |
| **EXPOSURE** |
| Ethnicity is a complex construct that groups people together who identify with each other through a shared culture, and encompasses factors in common such as cultural heritage, ancestry, language, history, diet, or religion. ^3^ Thus, it should be self-defined, according to the group the individual most identifies with.  Ethnicity is usually recorded by primary care staff during a patient’s initial registration with a practice. Primary care practices were financially incentivised to record ethnicity, under the UK’s Quality and Outcomes Framework (QOF), from 2006 to 2011. |
| **OUTCOMES** |
| **Derivation of proportion of days covered (PDC) and medication possession ratio (MPR)** |
| PDC was derived by dividing the number of days in the period “covered” (i.e. for which there was available medication) by the number of days in the period. ^4^ Overlapping prescription dates are not allowed, so PDC cannot exceed 100%.  MPR was derived by dividing the sum of the days’ supply in the period by the number of days in period, where the days’ supply in the period = quantity of tablets prescribed / the daily dose. ^4^  Where information on the quantity of tablets or daily dose was absent (N=272,890/2,694,313 prescriptions, 9%), we imputed the day’s supply for a particular prescription by using the median for the CPRD product code (which is specific to drug and dose).  The sample was limited to those with at least three prescriptions covering 90 days, excluding people with gaps between prescriptions exceeding six months and PDC/MPR under 20% or MPR exceeding 120% (likely to represent prescription errors). |
| **COVARIATES** |
| **Details of covariate derivation** |
| Gender was self-reported male or female (no other categories were present in the dataset). Quintilised Index of Multiple Deprivation (IMD) data, ^5^ designated by practice postcode, measured deprivation. Smoking was categorised as never, ex, or current, based on Read codes up to 5 years prior to and preferentially closest to the index date. SBP and DBP on or closest to the index date were used, along with BMI up to 5 years prior to and preferentially closest to the index date. Prevalent statin use and comorbidity (diabetes, chronic kidney disease [CKD, assigned by Read codes for stages 3-5 CKD and/ or eGFR≤ 60 ml/min/1.73m^2^, with a multiplier of 1.21 applied to eGFRs for people of African/ African Caribbean ethnicity], CVD [comprising coronary heart disease, stroke or peripheral vascular disease], cancer, asthma/chronic obstructive pulmonary disease [COPD] and serious mental illness) were determined at index date. The definition of diabetes for this study encompassed types 1, 2 and unspecified diabetes, as adjudicated by a previously published diagnostic algorithm, ^6^ since diabetes is inconsistently recorded in primary care records. Healthcare usage was categorised by tertile of the number of patient-initiated consultations in the year before the index date, and polypharmacy by tertile of the number of different medications prescribed in the year before the index date. |
| **STATISTICAL ANALYSIS** |
| **Details of missing data and justification of the complete case analysis approach** |
| Data were missing for ethnicity, smoking status and BMI (**figure S1**); variables which relied solely on physicians’ recording, and thus were not complete for all individuals. We chose a complete case analysis approach over multiple imputation as the missing at random assumption (necessary for multiple imputation) may not hold in primary care records (e.g., ethnicity ma y be more likely to be recorded in ethnicities other than white European, and thus its missingness is related to the ethnicity itself, BMI may more likely to be recorded if not in the healthy range ^7^ etc). Complete case analysis has been proven to be valid providing the missingness is conditionally independent of the outcome.^8^ |

**Table S2a). Baseline characteristics of antihypertensive-naïve people with at least one BP exceeding treatment threshold during the study period (2006-2019), by inclusion in the complete case analysis (based on missingness for covariates).** Data are n (age-standardised %) or age-adjusted mean±SD unless otherwise stated, complete case analysis. CKD=chronic kidney disease, COPD=chronic obstructive pulmonary disease.

|  | **Included in the complete case analysis** | **Excluded from the complete case analysis** |
| --- | --- | --- |
| **N (row %)** | 782,141 (89) | 100,718 (11) |
| **Ethnicity (row %):**  ***European***  ***South Asian***  ***African/ African Caribbean*** | 731,506 (87)  30,379 (90)  20,256 (89) | 96,060 (13)  2,746 (10)  1,912 (11) |
| **Age, yrs** | 51±15 | 50±19 |
| **Female gender** | 413,172 (56) | 42,272 (43) |
| **Index of Multiple deprivation quintile:**  ***1 (least deprived)***  ***2***  ***3***  ***4***  ***5 (most deprived)*** | 122,986 (16)  137,542 (18)  150,197 (19)  166,043 (21)  205,373 (26) | 17,296 (17)  15,591 (16)  20,164 (20)  21,719 (21)  25,569 (26) |
| **Cardiovascular disease risk factors** | | |
| **Smoking:**  ***Never***  ***Ex***  ***Current*** | 353,704 (46)  267,024 (33)  161,413 (20) | 50,329 (52)  25,419 (26)  22,406 (22) |
| **Systolic blood pressure (1^st^ reading):**  ***Mean ±SD, mmHg***  ***<140 mmHg***  ***140-149 mmHg***  ***150-159 mmHg***  ***160+ mmHg*** | 145±14  148,336 (21)  394,188 (51)  126,163 (15)  113,454 (18) | 148±15  14,635 (16)  48,943 (49)  17,553 (17)  19,587 (13) |
| **Diastolic blood pressure (1^st^ reading):**  ***Mean ±SD, mmHg***  ***80-89 mmHg***  ***90-99 mmHg***  ***100-110 mmHg***  ***110+ mmHg*** | 87±10  424,671 (56)  279,667 (35)  63,565 (7)  14,238 (2) | 87±11  52,227 (52)  35,245 (35)  10,262 (10)  2,984 (3) |
| **BMI, kg/m^2^** | 28±6 | 28±6 |
| **Statin use** | 71,393 (8) | 4,430 (4) |
| **Comorbidity** | | |
| **Diabetes** | 53,999 (7) | 2,257 (2) |
| **CKD** | 45,813 (7) | 3,757 (4) |
| **CVD** | 21,031 (4) | 3,218 (4) |
| **Serious mental illness** | 25,089 (3) | 2,029 (2) |
| **Cancer** | 32,993 (4) | 3,833 (4) |
| **Asthma/ COPD** | 94,807 (13) | 8,888 (9) |
| **Healthcare usage** | | |
| **No. consultations in previous year:**  ***Median (IQR)***  ***0-1 consultations***  ***2-5 consultations***  ***5 + consultations*** | 3 (1, 7)  229,990 (27)  280,803 (36)  271,348 (37) | 2 (0, 5)  42,735 (41)  33,243 (33)  24,740 (26) |
| **Polypharmacy** | | |
| **No. medications in previous year:**  ***Median (IQR)***  ***0 medications***  ***1-3 medications***  ***4 + medications*** | 1 (1, 2)  172,517 (20)  292,748 (37)  316,876 (43) | 1 (0, 2)  33,002 (31)  37,348 (37)  30,368 (32) |

**Table S2b). Baseline characteristics of antihypertensive-naïve people with at least one BP exceeding treatment threshold during the study period (2006-2019), by inclusion in the complete case analysis (based on missingness for ethnicity).** Data are n (age-standardised %) or age-adjusted mean±SD unless otherwise stated, complete case analysis. CKD=chronic kidney disease, COPD=chronic obstructive pulmonary disease. NB/ deprivation data not available for people with missing ethnicity.

|  | **Included in the complete case analysis** | | | **Excluded from the complete case analysis** |
| --- | --- | --- | --- | --- |
|  | **European ethnicity** | **South Asian ethnicity** | **African/ African Caribbean**  **ethnicity** | **Missing ethnicity** |
| **N (%)** | 731,506 (93.5) | 30,379 (3.9) | 20,256 (2.6) | 747,184 (100) |
| **Age, yrs** | 52±15 | 46±13 | 44±12 | 53±16 |
| **Female gender** | 387,619 (56) | 14,553 (49) | 11,000 (54) | 389,059 (55) |
| **Cardiovascular disease risk factors** | | | |  |
| **Smoking:**  ***Never***  ***Ex***  ***Current*** | 320,299 (45)  256,214 (34)  154,993 (21) | 20,286 (67)  6,363 (21)  3,730 (12) | 13,119 (65)  4,447 (23)  2,690 (12) | 350,725 (48)  242,006 (31)  154,453 (21) |
| **Systolic blood pressure (1^st^ reading):**  ***Mean ±SD, mmHg***  ***<140 mmHg***  ***140-149 mmHg***  ***150-159 mmHg***  ***160+ mmHg*** | 146±14  132,683 (20)  371,290 (51)  119,359 (15)  108,174 (14) | 143±14  10,438 (31)  13,336 (45)  3,763 (13)  2,842 (11) | 146±14  5,215 (24)  9,562 (46)  3,041 (16)  2,438 (14) | 147±14  116,849 (18)  374,042 (51)  127,757 (16)  128,536 (15) |
| **Diastolic blood pressure (1^st^ reading):**  ***Mean ±SD, mmHg***  ***80-89 mmHg***  ***90-99 mmHg***  ***100-110 mmHg***  ***110+ mmHg*** | 87±10  399,867 (56)  259,220 (35)  59,248 (7)  13,171 (2) | 87±9  15,167 (55)  12,344 (37)  2,312 (7)  556 (2) | 88±10  9,637 (54)  8,103 (35)  2,005 (8)  511 (2) | 89±10  404,116 (55)  261,152 (35)  66,148 (8)  15,768 (2) |
| **BMI, kg/m^2^** | 28±6 | 27±5 | 29±6 | 28±6 |
| **Statin use** | 65,802 (8) | 4,316 (15) | 1,275 (10) | 66,047 (7) |
| **Comorbidity** | | | |  |
| **Diabetes** | 46,140 (7) | 5,673 (19) | 2,186 (14) | 40,578 (6) |
| **CKD** | 43,234 (7) | 2,374 (9) | 205 (3) | 43,718 (6) |
| **CVD** | 20,357 (4) | 476 (3) | 198 (3) | 24,139 (4) |
| **Serious mental illness** | 23,553 (3) | 750 (2) | 786 (4) | 20,472 (3) |
| **Cancer** | 32,163 (5) | 483 (2) | 347 (4) | 34,664 (4) |
| **Asthma/ COPD** | 90,354 (13) | 2,897 (11) | 1,556 (9) | 138,341 (18) |
| **Healthcare usage** | | | |  |
| **No. consultations in previous year:**  ***Median (IQR)***  ***0-1 consultations***  ***2-5 consultations***  ***5 + consultations*** | 3 (1, 7)  216,766 (27)  262,721 (36)  252,019 (37) | 4 (2, 8)  7,543 (23)  10,542 (34)  12,294 (43) | 4 (1, 7)  5,681 (25)  7,540 (36)  7,035 (39) | 3 (1,7)  139,104 (17)  326,630 (43)  281,450 (40) |
| **Polypharmacy** | | | |  |
| **No. medications in previous year:**  ***Median (IQR)***  ***0 medications***  ***1-3 medications***  ***4 + medications*** | 1 (1, 2)  160,445 (20)  275,361 (37)  295,700 (43) | 1 (1, 2)  6,640 (20)  9,951 (30)  13,788 (50) | 1 (0, 2)  5,432 (24)  7,436 (33)  7,388 (43) | 1 (1,2)  115,313 (20)  224,293 (39)  226,513 (40) |

**Table S2c). Associations between ethnicity and hypertension management, antihypertensive use and BP control one year after antihypertensive initiation, by ethnicity and practice ethnicity recording status (above median % ethnicity recorded versus below median % ethnicity recorded).** Unless otherwise stated, data are OR, IRR or HR (95% CI), complete case analysis. All models are fully adjusted for: age, gender, SBP, deprivation, cardiovascular disease risk factors, comorbidity, healthcare usage and polypharmacy (plus antihypertensive class for adherence and persistence models; plus antihypertensive class, monitoring frequency, intensification and persistence and BP control models).

| **Sub-group** | **European ethnicity** | **South Asian ethnicity** | **African/ African Caribbean**  **ethnicity** |
| --- | --- | --- | --- |
| **Objective 1. BP re-measurement within three months of de novo raised BP** | | | |
| **Whole cohort** | 1 | 1.16 (1.09,1.24) | 0.98 (0.91,1.05) |
| **Practice ethnicity recording above median** | 1 | 1.14 (1.03,1.25) | 1.05 (0.96,1.14) |
| **Practice ethnicity recording below median** | 1 | 1.18 (1.09,1.28) | 0.94 (0.86,1.03) |
| **Objective 2. Guideline-indicated antihypertensive initiation within three months of second BP** | | | |
| **Whole cohort** | 1 | 1.49 (1.37,1.62) | 1.48 (1.32, 1.66) |
| **Practice ethnicity recording above median** | 1 | 1.62 (1.44,1.82) | 1.66 (1.41,1.95) |
| **Practice ethnicity recording below median** | 1 | 1.38 (1.22,1.57) | 1.35 (1.15,1.58) |
| **Objective 3a). BP monitoring rate in the first year of antihypertensive use** | | | |
| **Whole cohort** | 1 | 0.97 (0.94,1.00) | 0.91 (0.87,0.95) |
| **Practice ethnicity recording above median** | 1 | 0.96 (0.91,1.00) | 0.94 (0.88,1.01) |
| **Practice ethnicity recording below median** | 1 | 0.98 (0.94,1.02) | 0.88 (0.84,0.93) |
| **Objective 3b). Antihypertensive intensification within a year of above-target BP in the first year of use** | | | |
| **Whole cohort** | 1 | 1.10 (1.04,1.16) | 0.93 (0.87,1.00) |
| **Practice ethnicity recording above median** | 1 | 1.11 (1.03,1.20) | 0.99 (0.91,1.09) |
| **Practice ethnicity recording below median** | 1 | 1.09 (1.02,1.17) | 0.88 (0.81,0.97) |
| **Objective 4a). Persistence (no discontinuations exceeding three months) in the first year of antihypertensive use** | | | |
| **Whole cohort** | 1 | 0.48 (0.45,0.51) | 0.38 (0.35,0.42) |
| **Practice ethnicity recording above median** | 1 | 0.46 (0.43,0.50) | 0.37 (0.33,0.42) |
| **Practice ethnicity recording below median** | 1 | 0.50 (0.45,0.54) | 0.39 (0.35,0.44) |
| **Objective 4b). Adherence (proportion of days covered ≥80%) in the first year of use** | | | |
| **Whole cohort** | 1 | 0.51 (0.47,0.56) | 0.39 (0.36,0.43) |
| **Practice ethnicity recording above median** | 1 | 0.53 (0.47,0.60) | 0.41 (0.36,0.46) |
| **Practice ethnicity recording below median** | 1 | 0.49 (0.44,0.55) | 0.38 (0.33,0.43) |
| **Objective 5. BP control one year after antihypertensive initiation** | | | |
| **Whole cohort** | 1 | 1.09 (1.00,1.19) | 0.89 (0.81,0.98) |
| **Practice ethnicity recording above median** | 1 | 1.05 (0.93,1.20) | 0.81 (0.73,0.90) |
| **Practice ethnicity recording below median** | 1 | 1.13 (1.02,1.25) | 0.95 (0.83,1.08) |

**Table S2d). Baseline characteristics of people initiating antihypertensives, by missingness of adherence data.** Data are n (age-standardised %) or age-adjusted mean±SD unless otherwise stated, complete case analysis. CKD=chronic kidney disease, COPD=chronic obstructive pulmonary disease. NB/ deprivation data not available for people with missing ethnicity.

|  | **Adherence available** | **Adherence missing** |
| --- | --- | --- |
| **N (row %)** | 167,620 (83) | 33,559 (17) |
| **Ethnicity (row %):**  ***European***  ***South Asian***  ***African/ African Caribbean*** | 157,684 (84)  5,961 (76)  3,975 (74) | 30,189 (16)  1,868 (24)  1,502 (27) |
| **Age, yrs** | 60±13 | 58±15 |
| **Female gender** | 76,821 (48) | 17,030 (55) |
| **Index of Multiple deprivation quintile:**  ***1 (least deprived)***  ***2***  ***3***  ***4***  ***5 (most deprived)*** | 23,936 (13)  28,398 (16)  32,447 (19)  36,318 (22)  46,521 (30) | 4,937 (14)  5,527 (16)  6,440 (19)  7,333 (23)  9,322 (29) |
| **Cardiovascular disease risk factors** | | |
| **Smoking:**  ***Never***  ***Ex***  ***Current*** | 66,361 (43)  72,303 (38)  28,956 (19) | 13,541 (43)  13,692 (38)  6,326 (20) |
| **Systolic blood pressure (1^st^ reading):**  ***Mean ±SD, mmHg***  ***<140 mmHg***  ***140-149 mmHg***  ***150-159 mmHg***  ***160+ mmHg*** | 158±20  26,499 (19)  29,846 (20)  34,598 (19)  88,389 (42) | 152±20  8,613 (30)  7,712 (22)  6,699 (18)  13,151 (30) |
| **Diastolic blood pressure (1^st^ reading):**  ***Mean ±SD, mmHg***  ***80-89 mmHg***  ***90-99 mmHg***  ***100-110 mmHg***  ***110+ mmHg*** | 92±13  68,099 (34)  56,235 (31)  39,265 (24)  15,733 (11) | 89±13  16,686 (45)  10,453 (28)  6,642 (20)  2,394 (7) |
| **BMI, kg/m^2^** | 29±6 | 29±6 |
| **Statin use** | 56,664 (24) | 9,161 (20) |
| **Comorbidity** | | |
| **Diabetes** | 22,778 (15) | 4,032 (13) |
| **CKD** | 26,614 (15) | 5,796 (15) |
| **CVD** | 19,723 (9) | 3,131 (7) |
| **Serious mental illness** | 4,952 (3) | 1,192 (3) |
| **Cancer** | 11,768 (5) | 2,682 (6) |
| **Asthma/ COPD** | 22,223 (13) | 5,396 (16) |
| **Healthcare usage** | | |
| **No. consultations in previous year:**  ***Median (IQR)***  ***0-1 consultations***  ***2-5 consultations***  ***5 + consultations*** | 6 (3, 11)  44,272 (25)  62,988 (36)  60,360 (38) | 6 (3, 11)  9,097 (26)  11,804 (34)  12,658 (39) |
| **Polypharmacy** | | |
| **No. medications in previous year:**  ***Median (IQR)***  ***0 medications***  ***1-3 medications***  ***4 + medications*** | 4 (2, 8)  41,438 (25)  60,357 (36)  65,825 (40) | 4 (2, 9)  7,967 (24)  11,477 (35)  14,115 (41) |
| **First antihypertensive class initiated** | | |
| **ACE inhibitor/ angiotensin receptor blocker** | 91,885 (63) | 14,308 (44) |
| **Calcium channel blocker** | 58,565 (29) | 14,197 (41) |
| **Thiazide diuretic** | 16,948 (8) | 4,926 (14) |
| **Combination / other** | 222 (<1) | 128 (<1) |

**Table S3. Time-updated characteristics at antihypertensive initiation during the study period (2006-2019), by ethnicity.** Data are n (age-standardised %) or age-adjusted mean±SD unless otherwise stated, complete case analysis. CKD=chronic kidney disease, COPD=chronic obstructive pulmonary disease, ACE=angiotensin-converting enzyme.

|  | **European ethnicity** | **South Asian ethnicity** | **African/ African Caribbean**  **ethnicity** |
| --- | --- | --- | --- |
| **N (%)** | 187,873 (93) | 7,829 (4) | 5,477 (3) |
| **Age, yrs** | 60±13 | 53±13 | 50±11 |
| **Female gender** | 87,365 (49) | 3,590 (49) | 2,896 (53) |
| **Index of Multiple deprivation quintile:**  ***1 (least deprived)***  ***2***  ***3***  ***4***  ***5 (most deprived)*** | 27,896 (14)  32,450 (17)  36,428 (19)  39,551 (22)  51,548 (29) | 745 (9)  980 (11)  1,564 (20)  2,336 (30)  2,204 (29) | 232 (4)  495 (9)  895 (17)  1,764 (33)  2,091 (38) |
| **Cardiovascular disease risk factors** | | | |
| **Smoking:**  ***Never***  ***Ex***  ***Current*** | 71,487 (41)  82,528 (39)  33,858 (20) | 4,954 (64)  2,018 (25)  857 (11) | 3,461 (64)  1,449 (27)  567 (9) |
| **Systolic blood pressure (1^st^ reading):**  ***Mean ±SD, mmHg***  ***<140 mmHg***  ***140-149 mmHg***  ***150-159 mmHg***  ***160+ mmHg*** | 157±20  32,335 (21)  34,758 (20)  38,381 (19)  96,058 (40) | 153±20  1,892 (26)  1,694 (22)  1,654 (18)  2,959 (34) | 158±18  885 (19)  1,106 (19)  1,262 (21)  2,523 (42) |
| **Diastolic blood pressure (1^st^ reading):**  ***Mean ±SD, mmHg***  ***80-89 mmHg***  ***90-99 mmHg***  ***100-110 mmHg***  ***110+ mmHg*** | 91±13  80,221 (37)  62,223 (30)  42,452 (23)  16,636 (10) | 90±13  3,085 (39)  2,617 (30)  1,800 (21)  697 (10) | 92±12  1,479 (33)  1,848 (31)  1,655 (24)  794 (11) |
| **BMI, kg/m^2^** | 29±6 | 27±5 | 29±6 |
| **Statin use** | 61,866 (23) | 2,977 (32) | 982 (18) |
| **Comorbidity** | | | |
| **Diabetes** | 23,555 (14) | 2,354 (29) | 901 (17) |
| **CKD** | 31,002 (15) | 1,233 (15) | 175 (5) |
| **CVD** | 22,054 (8) | 624 (8) | 176 (5) |
| **Serious mental illness** | 5,788 (3) | 205 (3) | 151 (3) |
| **Cancer** | 14,066 (5) | 221 (3) | 163 (4) |
| **Asthma/ COPD** | 26,250 (16) | 909 (12) | 460 (10) |
| **Healthcare usage** | | | |
| **No. consultations in previous year:**  ***Median (IQR)***  ***0-1 consultations***  ***2-5 consultations***  ***5 + consultations*** | 6 (3, 11)  49,608 (25)  69,913 (36)  68,352 (39) | 7 (3, 11)  2,024 (24)  2,809 (36)  2,996 (40) | 6 (3, 10)  1,737 (29)  2,070 (35)  1,670 (36) |
| **Polypharmacy** | | | |
| **No. medications in previous year:**  ***Median (IQR)***  ***0 medications***  ***1-3 medications***  ***4 + medications*** | 4 (2, 8)  45,753 (24)  67,349 (36)  74,771 (40) | 5 (2, 10)  1,806 (21)  2,472 (32)  3,551 (47) | 3 (1, 6)  1,846 (29)  2,013 (36)  1,618 (35) |
| **First antihypertensive class initiated** | | | |
| **ACE inhibitor/ angiotensin receptor blocker** | 100,100 (60) | 4,830 (60) | 1,263 (24) |
| **Calcium channel blocker** | 66,562 (30) | 2,460 (33) | 3,740 (67) |
| **Thiazide diuretic** | 20,911 (9) | 503 (7) | 460 (9) |
| **Combination / other** | 300 (<1) | 36 (<1) | 14 (<1) |

**Table S4. Associations between ethnicity and hypertension management, antihypertensive use and BP control one year after antihypertensive initiation.** Unless otherwise stated, data are OR, IRR or HR (95% CI), complete case analysis. *Fully adjusted model factors: age, gender, SBP, deprivation, cardiovascular disease risk factors, comorbidity, healthcare usage and polypharmacy. Intensification data for nested cohort of individuals with a BP exceeding treatment target ≥ 30 days after antihypertensive initiation (N = 161,817). Adherence data for nested cohort of individuals with usable adherence measures (N = 167,620). BP control data for nested cohort with follow-up BP available at 12±6 months after antihypertensive initiation (N = 157,143).

| **Model factors** | **European ethnicity** | | **South Asian ethnicity** | **African/ African Caribbean**  **ethnicity** |
| --- | --- | --- | --- | --- |
| **Objective 1. BP re-measurement within three months of de novo raised BP** | | | | |
| **BP re-measured, n (%)** | 274,837 (34) | | 11,125 (34) | 7,132 (33) |
| **Age + gender** | 1 | | 1.07 (1.01,1.12) | 1.01 (0.95,1.07) |
| **Age + gender + SBP** | 1 | | 1.24 (1.17,1.32) | 1.03 (0.96,1.11) |
| **Fully-adjusted*** | 1 | | 1.16 (1.09,1.24) | 0.98 (0.91,1.05) |
| **Objective 2. Guideline-indicated antihypertensive initiation within three months of second BP** | | | | |
| **Antihypertensive initiation, n (%)** | 28,201 (23) | | 1,049 (20) | 823 (25) |
| **Age + gender** | 1 | | 0.95 (0.88,1.03) | 1.33 (1.21,1.47) |
| **Age + gender + SBP** | 1 | | 1.42 (1.31,1.55) | 1.49 (1.32,1.67) |
| **Fully-adjusted*** | 1 | | 1.49 (1.37,1.62) | 1.48 (1.32, 1.66) |
| **Objective 3a). BP monitoring rate in the first year of antihypertensive use** | | | | |
| **One or more BP measurement/s, n (%)** | 174,729 (93) | | 7,148 (91) | 4,958 (91) |
| **BP monitoring rate/ year, median (IQR)** | 3 (2,5) | | 3 (2,5) | 3 (2,5) |
| **Age + gender** | 1 | | 0.94 (0.91,0.97) | 0.93 (0.89,0.97) |
| **Age + gender + SBP** | 1 | | 0.98 (0.95,1.01) | 0.92 (0.89,0.96) |
| **Fully-adjusted*** | 1 | | 0.97 (0.94,1.00) | 0.91 (0.87,0.95) |
| **Objective 3b). Antihypertensive intensification within a year of above-target BP in the first year of use** | | | | |
| **Rate per 1000 PYAR (95% CI)** | 0.44(0.43,0.44) | | 0.41 (0.39,0.43) | 0.40 (0.38,0.43) |
| **Median (IQR) time to intensification** | 77 (30,174) | | 87 (36,179) | 84 (32,184) |
| **Age + gender** | 1 | | 0.96 (0.91,1.01) | 0.93 (0.87, 0.99) |
| **Age + gender + SBP** | 1 | | 1.03 (0.98,1.08) | 0.94 (0.88,1.00) |
| **Fully-adjusted*** | 1 | | 1.10 (1.04,1.16) | 0.93 (0.87,1.00) |
| **Objective 4a). Persistence (no discontinuations exceeding three months) in the first year of antihypertensive use** | | | | |
| **Persistent antihypertensive use, n (%)** | 171,402 (91) | 6,443 (82) | | 4,221 (77) |
| **Age + gender** | 1 | 0.49 (0.46, 0.52) | | 0.37 (0.34,0.40) |
| **Age + gender + SBP** | 1 | 0.47 (0.55,0.51) | | 0.37 (0.34,0.40) |
| **Age + gender + SBP + antihypertensive class** | 1 | 0.48 (0.45,0.51) | | 0.39 (0.36,0.42) |
| **Fully-adjusted* + antihypertensive class** | 1 | 0.48 (0.45,0.51) | | 0.38 (0.35,0.42) |
| **Objective 4b). Adherence (proportion of days covered ≥80%) in the first year of use** | | | | |
| **Antihypertensive adherent, n (%)** | 125,329 (79) | 3,777 (63) | | 2,248 (57) |
| **Age + gender** | 1 | 0.51 (0.45,0.56) | | 0.41 (0.37,0.44) |
| **Age + gender + SBP** | 1 | 0.52 (0.50,0.56) | | 0.41 (0.37,0.44) |
| **Age + gender + SBP + antihypertensive class** | 1 | 0.52 (0.48,0.56) | | 0.41 (0.38,0.44) |
| **Fully-adjusted*+ antihypertensive class** | 1 | 0.51 (0.47,0.56) | | 0.39 (0.36,0.43) |
| **Objective 5. BP control one year after antihypertensive initiation** | | | | |
| **BP ≤ target, n (%)** | 76,495 (52) | 3,087 (52) | | 1,855 (46) |
| **Age + gender** | 1 | 1.05 (0.97,1.14) | | 0.82 (0.74,0.89) |
| **Age + gender + SBP** | 1 | 0.98 (0.90,1.06) | | 0.81 (0.74,0.89) |
| **Age + gender + SBP + deprivation** | 1 | 0.98 (0.90,1.06) | | 0.81 (0.74,0.89) |
| **Age + gender + SBP + diabetes** | 1 | 1.07 (0.98,1.16) | | 0.82 (0.75,0.91) |
| **Age + gender + SBP + antihypertensive class** | 1 | 0.97 (0.90,1.05) | | 0.76 (0.69,0.83) |
| **Age + gender + SBP + monitoring frequency** | 1 | 0.98 (0.90,1.06) | | 0.81 (0.74,0.90) |
| **Age + gender + SBP + antihypertensive intensification** | 1 | 0.98 (0.90,1.06) | | 0.81 (0.73,0.89) |
| **Age + gender + SBP + persistence** | 1 | 1.05 (0.97,1.14) | | 0.91 (0.82,0.99) |
| **Fully-adjusted* + antihypertensive class + monitoring frequency + intensification + persistence** | 1 | 1.09 (1.00,1.19) | | 0.89 (0.81,0.98) |
| *Nested cohort with complete data for adherence* | | | | |
| **Age + gender** | 1 | 1.07 (0.98,1.17) | | 0.86 (0.79,0.96) |
| **Age + gender + SBP** | 1 | 1.01 (0.93,1.10) | | 0.86 (0.78,0.95) |
| **Age + gender + SBP + adherence** | 1 | 1.05 (0.96,1.15) | | 0.92 (0.83,1.01) |
| **Fully-adjusted* + antihypertensive class + monitoring frequency + intensification + persistence** | 1 | 1.13 (1.04,1.23) | | 0.93 (0.85,1.03) |
| *Subgroup analysis by antihypertensive adherence* | | | | |
| **Fully-adjusted* + antihypertensive class + monitoring frequency + intensification + persistence:**  Adherent  Non- adherent | 1  1 | 1.15 (1.04,1.27)  1.10 (0.99,1.23)  p interaction = 0.55 | | 0.99 (0.88,1.10)  0.88 (0.77,0.99)  p interaction = 0.07 |

**Table S5.** **Sub-group analysis of associations between ethnicity and BP control one year after antihypertensive initiation, by persistence/ adherence crossed categories.** Unless otherwise stated, data are n (within ethnicity %), OR (95% CI), complete case analysis. Models adjusted for: age, gender, SBP, deprivation, cardiovascular disease risk factors, comorbidity, healthcare usage, polypharmacy, antihypertensive class, monitoring frequency and intensification. Data from nested cohort of individuals with usable adherence measures (N = 167,620).

|  | **European ethnicity**  **N = 157,684** | **South Asian ethnicity**  **N = 5,961** | **African/ African Caribbean**  **ethnicity**  **N = 3,975** |
| --- | --- | --- | --- |
| **Non-persistent + non-adherent** | 9,673 (6)  1 (reference) | 869 (15)  1.11 (0.93,1.31) | 781 (20)  0.82 (0.67,1.01) |
| **Non-persistent + adherent** | 2,258 (1)  1 (reference) | 105 (2)  1.14 (0.71,1.83) | 69 (2)  1.09 (0.66,1.78) |
| **Persistent + non-adherent** | 22,682 (14)  1 (reference) | 1,315 (22)  1.10 (0.95,1.26) | 946 (24)  0.93 (0.80,1.09) |
| **Persistent + adherent** | 123,071 (78)  1 (reference) | 3,672 (62)  1.15 (1.04,1.27) | 2,179 (55)  0.98 (0.87,1.09) |

**Table S6. Associations between ethnicity and hypertension management, antihypertensive use and BP control one year after antihypertensive initiation: sub-group analysis by diabetes status.** Unless otherwise stated, data are OR, IRR or HR (95% CI), complete case analysis. *Fully adjusted model factors: age, gender, SBP, deprivation, cardiovascular disease risk factors, comorbidity, healthcare usage and polypharmacy. Intensification data for nested cohort of individuals with a BP exceeding treatment target ≥ 30 days after antihypertensive initiation (N = 161,817). Adherence data for nested cohort of individuals with usable adherence measures (N = 167,620). BP control data for nested cohort with follow-up BP available at 12±6 months after antihypertensive initiation (N = 157,143).

| **Model factors** | **European ethnicity** | | **South Asian ethnicity** | **African/ African Caribbean**  **ethnicity** |
| --- | --- | --- | --- | --- |
| **Objective 1. BP re-measurement within three months of de novo raised BP** | | | | |
| **People without diabetes** | | | | |
| **BP re-measured, n (%)** | 258,788 (34) | | 9,158 (34) | 6,390 (33) |
| **Age + gender** | 1 | | 1.07 (1.01,1.13) | 1.01 (0.95,1.08) |
| **Fully-adjusted*** | 1 | | 1.14 (1.07,1.22) | 0.97 (0.90,1.04) |
| **People with diabetes** | | | | |
| **BP re-measured, n (%)** | 16,049 (34) | | 1,967 (35) | 742 (33) |
| **Age + gender** | 1 | | 1.03 (0.96,1.11)  p interaction = 0.38 | 0.99 (0.88,1.12)  p interaction = 0.78 |
| **Fully-adjusted*** | 1 | | 1.27 (1.17,1.38)  p interaction = 0.02 | 1.10 (0.96,1.26)  p interaction = 0.05 |
| **Objective 2. Guideline-indicated antihypertensive initiation within three months of second BP** | | | | |
| **People without diabetes** | | | | |
| **Antihypertensive initiation, n (%)** | 26,161 (24) | | 819 (24) | 720 (28) |
| **Age + gender** | 1 | | 1.14 (1.04,1.25) | 1.46 (1.31,1.63) |
| **Fully-adjusted*** | 1 | | 1.44 (1.31,1.58) | 1.58 (1.42,1.77) |
| **People with diabetes** | | | | |
| **Antihypertensive initiation, n (%)** | 2,040 (13) | | 230 (12) | 103 (14) |
| **Age + gender** | 1 | | 0.97 (0.83,1.14)  p interaction = 0.10 | 1.16 (0.94,1.45)  p interaction = 0.06 |
| **Fully-adjusted*** | 1 | | 1.33 (1.14,1.56)  p interaction = 0.42 | 1.31 (1.03,1.65)  p interaction = 0.13 |
| **Objective 3a). BP monitoring in the first year of antihypertensive use** | | | | |
| **People without diabetes** | | | | |
| **One or more BP measurement/s, n (%)** | 152,657 (93) | | 5,001 (91) | 4,146 (91) |
| **BP monitoring rate/ year, median (IQR)** | 3 (2,5) | | 3 (2,5) | 3 (2,5) |
| **Age + gender** | 1 | | 0.96 (0.93,1.00) | 0.94 (0.90,0.99) |
| **Fully-adjusted*** | 1 | | 0.98 (0.94,1.01) | 0.92 (0.88,0.96) |
| **People with diabetes** | | | | |
| **One or more BP measurement/s, n (%)** | 22,072 (94) | | 2,147 (91) | 812 (90) |
| **BP monitoring rate/ year, median (IQR)** | 3 (2,5) | | 3 (1,5) | 3 (1,4) |
| **Age + gender** | 1 | | 0.92 (0.88,0.97)  p interaction = 0.06 | 0.88 (0.82,0.93)  p interaction = 0.02 |
| **Fully-adjusted*** | 1 | | 0.95 (0.91,1.00)  p interaction = 0.27 | 0.87 (0.81,0.92)  p interaction = 0.07 |
| **Objective 3b). Antihypertensive intensification within a year of above-target BP in the first year of use** | | | | |
| **People without diabetes** | | | | |
| **Rate per 1000 PYAR (95% CI)** | 0.46(0.46,0.47) | | 0.47 (0.45,0.50) | 0.42 (0.39,0.44) |
| **Median (IQR) time to intensification** | 76 (29,170) | | 85 (35,169) | 78 (31,176) |
| **Age + gender** | 1 | | 1.02 (0.96,1.07) | 0.91 (0.84, 0.98) |
| **Fully-adjusted*** | 1 | | 1.08 (1.02,1.14) | 0.89 (0.83,0.96) |
| **People with diabetes** | | | | |
| **Rate per 1000 PYAR (95% CI)** | 0.28 (0.27,0.28) | | 0.30 (0.27,0.33) | 0.34 (0.29,0.39) |
| **Median (IQR) time to intensification** | 97 (35,203) | | 92 (43,198) | 112 (36,244) |
| **Age + gender** | 1 | | 1.07 (0.97,1.18)  p interaction = 0.35 | 1.20 (1.04,1.39)  p interaction = 0.001 |
| **Fully-adjusted*** | 1 | | 1.18 (1.07,1.30)  p interaction = 0.09 | 1.22 (1.06,1.40)  p interaction < 0.001 |
| **Objective 4a). Persistence (no discontinuations exceeding three months) in the first year of antihypertensive use** | | | | |
| **People without diabetes** | | | | |
| **Persistent antihypertensive use, n (%)** | 149,984 (91) | 4,503 (82) | | 3,493 (76) |
| **Age + gender** | 1 | 0.49 (0.46, 0.52) | | 0.35 (0.32,0.39) |
| **Fully-adjusted* + antihypertensive class** | 1 | 0.48 (0.45,0.51) | | 0.37 (0.34,0.41) |
| **People with diabetes** | | | | |
| **Persistent antihypertensive use, n (%)** | 21,418 (91) | 1,940 (82) | | 728 (81) |
| **Age + gender** | 1 | 0.50 (0.44,0.56)  p interaction = 0.74 | | 0.45 (0.37,0.54)  p interaction = 0.02 |
| **Fully-adjusted* + antihypertensive class** | 1 | 0.49 (0.43,0.55)  p interaction = 0.77 | | 0.46 (0.38,0.56)  p interaction = 0.04 |
| **Objective 4b). Adherence (proportion of days covered ≥80%) in the first year of use** | | | | |
| **People without diabetes** | | | | |
| **Antihypertensive adherent, n (%)** | 109,560 (80) | 2,646 (64) | | 1,843 (56) |
| **Age + gender** | 1 | 0.52 (0.48,0.57) | | 0.40 (0.37,0.44) |
| **Fully-adjusted*+ antihypertensive class** | 1 | 0.52 (0.47,0.56) | | 0.39 (0.36,0.43) |
| **People with diabetes** | | | | |
| **Antihypertensive adherent, n (%)** | 15,769 (78) | 1,131 (62) | | 405 (58) |
| **Age + gender** | 1 | 0.52 (0.46,0.58)  p interaction = 0.88 | | 0.43 (0.37,0.51)  p interaction = 0.46 |
| **Fully-adjusted*+ antihypertensive class** | 1 | 0.51 (0.45,0.58)  p interaction = 0.87 | | 0.41 (0.35,0.49)  p interaction = 0.51 |
| **Objective 5. BP control one year after antihypertensive initiation** | | | | |
| **People without diabetes** | | | | |
| **BP ≤ target, n (%)** | 67,782 (53) | 2,196 (55) | | 1,574 (47) |
| **Age + gender** | 1 | 1.11 (1.02,1.21) | | 0.82 (0.74,0.90) |
| **Age + gender + SBP + persistence** | 1 | 1.20 (1.10,1.31) | | 0.93 (0.84,1.02) |
| **Fully-adjusted* + antihypertensive class + monitoring frequency + intensification + persistence** | 1 | 1.10 (1.01,1.20) | | 0.89 (0.81,0.98) |
| *Sub-group analysis by antihypertensive adherence* | | | | |
| **Fully-adjusted* + antihypertensive class + monitoring frequency + intensification + persistence:**  Adherent  Non- adherent | 1  1 | 1.16 (1.02,1.30)  1.12 (0.99,1.27)  p interaction = 0.63 | | 0.98 (0.87,1.11)  0.88 (0.76,1.00)  p interaction = 0.16 |
| **People with diabetes** | | | | |
| **BP ≤ target, n/N (%)** | 8,713 (44) | 891 (47) | | 281 (40) |
| **Age + gender** | 1 | 1.14 (1.01,1.27)  p interaction = 0.66 | | 0.86 (0.71,1.02)  p interaction = 0.65 |
| **Age + gender + SBP + persistence** | 1 | 1.21 (1.08,1.36)  p interaction = 0.83 | | 0.92 (0.77,1.10)  p interaction = 0.95 |
| **Fully-adjusted* + antihypertensive class + monitoring frequency + intensification + persistence** | 1 | 1.07 (0.95,1.21)  p interaction = 0.71 | | 0.88 (0.73,1.05)  p interaction = 0.90 |
| *Sub-group analysis by antihypertensive adherence* | | | | |
| **Fully-adjusted* + antihypertensive class + monitoring frequency + intensification + persistence:**  Adherent  Non- adherent | 1  1 | 1.16 (1.00,1.35)  1.10 (0.92,1.32)  p interaction = 0.59 | | 1.00 (0.80,1.25)  0.83 (0.62,1.12)  p interaction = 0.30 |

**Table S7.** **Associations between ethnicity and hypertension management, antihypertensive use and BP control one year after antihypertensive initiation: sub-group analysis by calendar time period.** Unless otherwise stated, data are n(%), median (IQR) or rate per 1000 PYAR (95% CI) for objective 3b), on the top line of the cell, followed by OR, IRR or HR (95% CI) on the bottom line, ethnicity x time period interaction from likelihood ratio test, complete case analysis. Models are fully adjusted for: age, gender, SBP, deprivation, cardiovascular disease risk factors, comorbidity, healthcare usage and polypharmacy. Intensification data for nested cohort of individuals with a BP exceeding treatment target ≥ 30 days after antihypertensive initiation (N = 161,817). Adherence data for nested cohort of individuals with usable adherence measures (N = 167,620). BP control data for nested cohort with follow-up BP available at 12±6 months after antihypertensive initiation (N = 157,143).

| **Calendar period** | **European ethnicity** | **South Asian ethnicity** | **African/ African**  **Caribbean ethnicity** | **Ethnicity x time period interaction p** |
| --- | --- | --- | --- | --- |
| **Objective 1. BP re-measurement within three months of de novo raised BP** | | | | |
| 1st Jan 2006 to 31st Aug 2011 | 156,577 (36)  1 | 5,340 (36)  1.16 (1.09,1.24) | 3,472 (35)  0.98 (0.91,1.06) | 0.09 |
| 1^st^ Sept 2011 to 31^st^ Jan 2015 | 54,578 (33)  1 | 2,962 (35)  1.23 (1.15,1.31) | 1,962 (33)  1.01 (0.93,1.10) |  |
| 1st Feb 2015 to 30th June 2019 | 33,830 (31)  1 | 1,936 (31)  1.11 (0.94,1.32) | 1,134 (30)  0.97 (0.88,1.08) |  |
| **Objective 2. Guideline-indicated antihypertensive initiation within three months of second BP** | | | | |
| 1st Jan 2006 to 31st Aug 2011 | 20,556 (25)  1 | 592 (22)  1.43 (1.27,1.61) | 468 (27)  1.46 (1.26,1.70) | 0.87 |
| 1st Sept 2011 to 31st Jan 2015 | 4,819 (19)  1 | 277 (18)  1.61 (1.4,1.87) | 214 (23)  1.56 (1.31,1.87) |  |
| 1st Feb 2015 to 30th June 2019 | 2,826 (17)  1 | 180 (17)  1.64 (1.32,2.04) | 141 (23)  1.48 (1.18,1.87) |  |
| **Objective 3a). BP monitoring rate in the first year of antihypertensive use** | | | | |
| 1st Jan 2006 to 31st Aug 2011 | 4 (2,6)  1 | 4 (2,6)  1.00 (0.96,1.04) | 4 (2,6)  0.95 (0.91,0.99) | 0.03 |
| 1^st^ Sept 2011 to 31^st^ Jan 2015 | 3 (2,5)  1 | 3 (2,5)   - 1. (0.96,1.04) | 3 (2,5)  0.92 (0.88,0.97) |  |
| 1st Feb 2015 to 30th June 2019 | 2 (1,4)  1 | 2 (1,4)  0.94 (0.89,1.00) | 2 (1,4)  0.90 (0.85,0.96) |  |
| **Objective 3b). Antihypertensive intensification within a year of above-target BP in the first year of use** | | | | |
| 1st Jan 2006 to 31st Aug 2011 | 0.44 (0.43,0.44)  1 | 0.41 (0.38,0.44)  1.10 (1.04,1.19) | 0.42 (0.39,0.45)  0.99 (0.91,1.09) | 0.34 |
| 1st Sept 2011 to 31st Jan 2015 | 0.43 (0.42,0.43)  1 | 0.42 (0.39,0.46)  1.13 (1.03,1.23) | 0.38 (0.35,0.42)  0.89 (0.81,0.98) |  |
| 1st Feb 2015 to 30th June 2019 | 0.45 (0.44,0.46)  1 | 0.42 (0.37,0.46)  1.05 (0.92,1.19) | 0.40 (0.35,0.45)  0.86 (0.74,0.99) |  |
| **Objective 4a). Persistence (no discontinuations exceeding three months) in the first year of antihypertensive use** | | | | |
| 1st Jan 2006 to 31st Aug 2011 | 84,442 (90)  1 | 2,672 (79)  0.42 (0.39,0.46) | 1,682 (74)  0.36 (0.32,0.41) | 0.08 |
| 1st Sept 2011 to 31st Jan 2015 | 47,562 (91)  1 | 1,971 (83)  0.52 (0.46,0.59) | 1,453 (77)  0.41 (0.36,0.46) |  |
| 1^st^ Feb 2015 to 30^th^ June 2019 | 39,398 (93)  1 | 1,800 (87)  0.54 (0.46,0.63) | 1,086 (81)  0.40 (0.34,0.47) |  |
| **Objective 4b). Adherence (proportion of days covered ≥80%) in the first year of use** | | | | |
| 1st Jan 2006 to 31st Aug 2011 | 65,888 (81)  1 | 1,682 (63)  0.45 (0.41,0.50) | 973 (57)  0.38 (0.33,0.43) | <0.001 |
| 1st Sept 2011 to 31st Jan 2015 | 33,468 (77)  1 | 1,128 (61)  0.56 (0.49,0.64) | 753 (54)  0.40 (0.36,0.46) |  |
| 1st Feb 2015 to 30th June 2019 | 25,973 (79)  1 | 967 (66)  0.61 (0.53,0.70) | 522 (58)  0.43 (0.37,0.50) |  |
| **Objective 5. BP control one year after antihypertensive initiation** | | | | |
| 1st Jan 2006 to 31st Aug 2011 | 36,056 (44)  1 | 1,241 (43)  0.99 (0.88,1.11) | 656 (35)  0.76 (0.68,0.85) | 0.07 |
| 1st Sept 2011 to 31st Jan 2015 | 25,383 (63)  1 | 1,082 (61)  1.01 (0.95,1.16) | 792 (56)  0.95 (0.84,1.08) |  |
| 1st Feb 2015 to 30th June 2019 | 15,056 (62)  1 | 764 (63)  1.24 (1.08,1.43) | 407 (54)  0.91 (0.78,1.07) |  |

**Table S8. Sensitivity analyses of associations between ethnicity and guideline-indicated antihypertensive initiation.** Data are OR (95% CI) unless otherwise stated, complete case analysis. *Fully adjusted model factors: age, gender, SBP, deprivation, cardiovascular disease risk factors, comorbidity, healthcare usage and polypharmacy.

| **Model factors** | **European ethnicity** | **South Asian ethnicity** | **African/ African Caribbean**  **ethnicity** |
| --- | --- | --- | --- |
| **a) Using a six month (instead of three month) cut-point for antihypertensive initiation** | | | |
| **Antihypertensive initiation, n (%)** | 31,523 (25) | 1,192 (23) | 921 (28) |
| **Age + gender** | 1 | 0.97 (0.90,1.04) | 1.35 (1.22,1.48) |
| **Age + gender + SBP** | 1 | 1.44 (1.33,1.56) | 1.51 (1.34,1.69) |
| **Fully-adjusted*** | 1 | 1.50 (1.38,1.62) | 1.49 (1.33, 1.67) |
| **b) Using ambulatory BP monitoring Read codes for the second BP** | | | |
| **Antihypertensive initiation, n (%)** | 476 (30) | 19 (30) | 14 (40) |
| **Age + gender** | 1 | 1.01 (0.61,1.68) | 1.55 (0.89,2.71) |
| **Age + gender + SBP** | 1 | 1.44 (0.75,2.74) | 1.48 (0.77,2.83) |
| **c) Using clinic BP thresholds (instead of ambulatory BP thresholds) for the second BP to derive the denominator for treatment for records from 2011-2019** | | | |
| **Antihypertensive initiation, n (%)** | 27,695 (23) | 1,024 (20) | 793 (26) |
| **Age + gender** | 1 | 0.94 (0.87,1.02) | 1.34 (1.21,1.48) |
| **Age + gender + SBP** | 1 | 1.42 (1.31,1.54) | 1.48 (1.32,1.67) |
| **Fully-adjusted*** | 1 | 1.50 (1.37,1.63) | 1.48 (1.32,1.67) |

**Table S9. Sensitivity analyses of associations between ethnicity and antihypertensive use in the first year after antihypertensive initiation: a) defining persistence by lack of discontinuations exceeding six (instead of three) months and b) defining adherence by medication possession ratio (instead of proportion of days covered).** Data are OR (95% CI) unless otherwise stated, complete case analysis. *Fully adjusted model factors: age, gender, SBP, deprivation, cardiovascular disease risk factors, comorbidity, healthcare usage, polypharmacy and initial antihypertensive class. Adherence data for nested cohort of individuals with usable adherence measures (N = 167,620).

| **Model factors** | **European ethnicity** | **South Asian ethnicity** | **African/ African Caribbean**  **ethnicity** |
| --- | --- | --- | --- |
| **Persistence (no discontinuations exceeding six months) in the first year of antihypertensive use** | | | |
| **Persistent antihypertensive use, n (%)** | 180,827 (96) | 7,273 (93) | 4,952 (90) |
| **Age + gender** | 1 | 0.55 (0.49,0.60) | 0.40 (0.36,0.45) |
| **Age + gender + SBP** | 1 | 0.53 (0.48,0.59) | 0.41 (0.37,0.45) |
| **Age + gender + SBP + antihypertensive class** | 1 | 0.53 (0.48,0.59) | 0.44 (0.40,0.49) |
| **Fully-adjusted*** | 1 | 0.53 (0.48,0.59) | 0.44 (0.40,0.49) |
| **Sub-group analysis by diabetes status (fully adjusted model):**  ***No diabetes***  ***Diabetes***  ***Ethnicity x diabetes interaction p-value*** | 1  1  - | 0.54 (0.48, 0.61)  0.50 (0.41,0.61)  0.53 | 0.42 (0.38,0.47)  0.54 (0.41,0.71)  0.11 |
| **Adherence (medication possession ratio ≥ 80%) in the first year of antihypertensive use** | | | |
| **Antihypertensive adherent, n (%)** | 124,058 (86) | 3,962 (73) | 2,471 (67) |
| **Age + gender** | 1 | 0.54 (0.49,0.59) | 0.42 (0.38,0.46) |
| **Age + gender + SBP** | 1 | 0.55 (0.50,0.61) | 0.41 (0.37,0.46) |
| **Age + gender + SBP + antihypertensive class** | 1 | 0.55 (0.50,0.60) | 0.41 (0.38,0.46) |
| **Fully-adjusted*** | 1 | 0.54 (0.49,0.60) | 0.39 (0.36,0.43) |
| **Sub-group analysis by diabetes status (fully adjusted model):**  ***No diabetes***  ***Diabetes***  ***Ethnicity x diabetes interaction p-value*** | 1  1  - | 0.52 (0.47,0.58)  0.60 (0.52,0.69)  0.05 | 0.39 (0.35,0.43)  0.42 (0.35,0.51)  0.42 |

**Table S10.** **Sensitivity analysis of associations between ethnicity and hypertension management and BP control one year after antihypertensive initiation, using BP thresholds in operation at the study outset.** Unless otherwise stated, data are OR (95% CI), complete case analysis. *Fully adjusted model factors: age, gender, SBP, deprivation, cardiovascular disease risk factors, comorbidity, healthcare usage and polypharmacy. BP control data for nested cohort with follow-up BP available at 12±6 months after antihypertensive initiation (N = 157,143).

| **Model factors** | **European ethnicity** | | **South Asian ethnicity** | **African/ African Caribbean**  **ethnicity** |
| --- | --- | --- | --- | --- |
| **Objective 1. BP re-measurement within three months of de novo raised BP** | | | | |
| **BP re-measured, n (%)** | 243,231 (35) | | 9,803 (36) | 6,436 (34) |
| **Age + gender** | 1 | | 1.10 (1.05,1.17) | 1.03 (0.96,1.09) |
| **Age + gender + SBP** | 1 | | 1.22 (1.15,1.30) | 1.03 (0.95,1.10) |
| **Fully-adjusted*** | 1 | | 1.16 (1.08,1.23) | 0.98 (0.91,1.05) |
| **Objective 2. Guideline-indicated antihypertensive initiation within three months of second BP** | | | | |
| **Antihypertensive initiation, n (%)** | 28,292 (23) | | 1,037 (21) | 826 (26) |
| **Age + gender** | 1 | | 1.01 (0.93,1.09) | 1.37 (1.25,1.51) |
| **Age + gender + SBP** | 1 | | 1.39 (1.28,1.52) | 1.48 (1.32,1.66) |
| **Fully-adjusted*** | 1 | | 1.45 (1.33,1.58) | 1.47 (1.31, 1.65) |
| **Objective 5. BP control one year after antihypertensive initiation** | | | | |
| **BP ≤ target, n (%)** | 64,539 (42) | 2,384 (41) | | 1,490 (36) |
| **Age + gender** | 1 | 0.97 (0.90,1.04) | | 0.81 (0.74,0.89) |
| **Age + gender + SBP** | 1 | 0.92 (0.86,1.00) | | 0.81 (0.73,0.89) |
| **Age + gender + SBP + diabetes** | 1 | 1.02 (0.95,1.10) | | 0.82 (0.75,0.90) |
| **Age + gender + SBP + antihypertensive class** | 1 | 0.92 (0.86,1.00) | | 0.77 (0.69,0.84) |
| **Age + gender + SBP + monitoring frequency** | 1 | 0.92 (0.86,1.01) | | 0.81 (0.74,0.89) |
| **Age + gender + SBP + antihypertensive intensification** | 1 | 0.92 (0.87,1.01) | | 0.81 (0.73,0.88) |
| **Age + gender + SBP + persistence** | 1 | 0.98 (0.91,1.06) | | 0.89 (0.81,0.98) |
| **Fully-adjusted* + antihypertensive class + monitoring frequency + intensification + persistence** | 1 | 1.03 (0.96,1.12) | | 0.83 (0.76,0.91) |
| *Nested cohort with complete data for adherence* | | | | |
| **Age + gender** | 1 | 0.99 (0.92,1.07) | | 0.86 (0.77,0.95) |
| **Age + gender + SBP** | 1 | 0.95 (0.88,1.03) | | 0.85 (0.77,0.94) |
| **Age + gender + SBP + adherence** | 1 | 0.99 (0.92,1.07) | | 0.91 (0.82,1.00) |
| **Fully-adjusted* + antihypertensive class + monitoring frequency + intensification + persistence** | 1 | 1.07 (1.00,1.17) | | 0.88 (0.77,0.97) |
| *Subgroup analysis by antihypertensive adherence* | | | | |
| **Fully-adjusted* + antihypertensive class + monitoring frequency + intensification + persistence:**  Adherent  Non- adherent | 1  1 | 1.07 (0.98,1.19)  1.06 (0.95,1.19)  p interaction = 0.82 | | 0.89 (0.79,1.00)  0.86 (0.76,0.97)  p interaction = 0.65 |

**Table S11. Sub-group analyses of persistence and adherence by i) age group, ii) gender, iii) deprivation and iv) antihypertensive class.** Fully adjusted models adjusted for age, gender, ethnicity, SBP, deprivation, cardiovascular disease risk factors, comorbidity, healthcare usage, polypharmacy and antihypertensive class, complete case analysis. ACE = angiotensin converting enzyme.

| **Model factors** | **n (%)** | **Fully adjusted* OR (95% CI)** |
| --- | --- | --- |
| **PERSISTENT ANTIHYPERTENSIVE USE** | | |
| **i) Sub-group analysis by age** | | |
| **< 40 years old** | 11,946 (86) | 0.80 (0.66,0.95) |
| **40 to 49 years old** | 32,322 (88) | 0.88 (0.76, 1.01) |
| **50 to 59 years old** | 49,729 (90) | 1.03 (0.93,1.14) |
| **60 to 69 years old** | 52,082 (92) | 1.17 (1.09,1.25) |
| **70+ years old** | 48,910 (92) | 1 |
| **ii) Sub-group analysis by gender** | | |
| **Female** | 90,558 (91) | 1 |
| **Male** | 104,431 (90) | 0.91 (0.88,0.94) |
| **iii) Sub-group analysis by deprivation quintile** | | |
| **Least deprived quintile** | 28,571 (91) | 1 |
| **Second least deprived quintile** | 32,806 (91) | 1.01 (0.93,1.10) |
| **Median quintile** | 38,087 (91) | 1.01 (0.94,1.10) |
| **Second most deprived quintile** | 42,165 (90) | 0.96 (0.88,1.05) |
| **Most deprived quintile** | 53,330 (90) | 0.94 (0.88,1.01) |
| **iv) Sub-group analysis by antihypertensive class** | | |
| **ACE inhibitor/ Angiotensin receptor blocker** | 103,042 (91) | 1 |
| **Calcium channel blocker** | 70,276 (90) | 0.87 (0.84,0.90) |
| **Thiazide diuretic** | 21,338 (90) | 0.78 (0.74,0.83) |
| **ADHERENT TO ANTIHYPERTENSIVES** | | |
| **i) Sub-group analysis by age** | | |
| **< 40 years old** | 6,234 (63) | 0.61 (0.52,0.71) |
| **40 to 49 years old** | 21,560 (72) | 0.78 (0.69,0.87) |
| **50 to 59 years old** | 35,711 (77) | 0.90 (0.83,0.97) |
| **60 to 69 years old** | 40,397 (83) | 1.15 (1.09,1.21) |
| **70+ years old** | 36,668 (83) | 1 |
| **ii) Sub-group analysis by gender** | | |
| **Female** | 64,687 (79) | 1 |
| **Male** | 75,883 (78) | 0.91 (0.89,0.94) |
| **iii) Sub-group analysis by deprivation quintile** | | |
| **Least deprived quintile** | 20,424 (79) | 1 |
| **Second least deprived quintile** | 23,650 (79) | 1.01 (0.80,1.28) |
| **Median quintile** | 27,379 (78) | 1.03 (0.82,1.28) |
| **Second most deprived quintile** | 30,475 (78) | 1.07 (0.86,1.32) |
| **Most deprived quintile** | 38,636 (78) | 1.08 (0.90,1.29) |
| **iv) Sub-group analysis by antihypertensive class** | | |
| **ACE inhibitor/ Angiotensin receptor blocker** | 75,857 (77) | 1 |
| **Calcium channel blocker** | 49,767 (79) | 0.96 (0.91,1.01) |
| **Thiazide diuretic** | 14,757 (80) | 0.90 (0.83,0.97) |

**Table S12. Ethnic differences in hypertension management, antihypertensive usage in the first year of treatment and blood pressure control one year after antihypertensive initiation, by ethnic sub-group.** Data are N with available data or HR/ IRR/ OR (95% CI) adjusted for age, gender, systolic blood pressure, deprivation, cardiovascular disease risk factors, comorbidity, healthcare usage and polypharmacy. Adherence/ discontinuation models also adjusted for antihypertensive class. BP control models also adjusted for antihypertensive class, monitoring frequency, intensification and discontinuation. Complete case analysis.

| **Ethnic sub-group**  *N (%)* | **BP re-measurement** | **Anti-hypertensive initiation** | **N initiating anti-hypertensives** | **BP monitoring** | **Anti-hypertensive intensification** | **Anti-hypertensive persistence** | **Anti-hypertensive adherence** | **BP control** |
| --- | --- | --- | --- | --- | --- | --- | --- | --- |
| **British**  *689,405 (87.9)* | 1 | 1 | 179,439 (88.9) | 1 | 1 | 1 | 1 | 1 |
| **Irish**  *7,362 (0.9)* | 1.01 (0.96,1.05) | 1.00 (0.90,1.10) | 1,853 (0.9) | 1.00 (0.96,1.04) | 0.97 (0.88,1.07) | 0.88  (0.80, 0.98) | 0.74 (0.62,0.89) | 0.96 (0.84,1.09) |
| **Other white**  *36,420 (4.6)* | 0.98 (0.96,1.00) | 1.06 (0.99,1.13) | 7,203 (3.6) | 0.87 (0.84,0.90) | 0.97 (0.93,1.03) | 0.58 (0.53,0.64) | 0.66 (0.56,0.76) | 0.97 (0.91,1.04) |
| **Indian**  *13,002 (1.7)* | 1.08 (1.02,1.15) | 1.24 (1.14,1.35) | 3,325 (1.7) | 0.95 (0.91,0.99) | 1.05 (0.97,1.14) | 0.54 (0.50,0.59) | 0.48 (0.42,0.56) | 1.11 (1.01,1.22) |
| **Pakistani**  *6,732 (0.9)* | 1.10 (1.02,1.19) | 1.29 (1.13,1.47) | 1,732 (0.9) | 0.99 (0.95,1.03) | 1.17 (1.05,1.30) | 0.57 (0.51,0.65) | 0.44 (0.37,0.53) | 1.11 (0.96,1.28) |
| **Bangladeshi**  *2,375 (0.3)* | 1.23 (1.13,1.34) | 1.67 (1.41,1.99) | 654 (0.3) | 1.07 (0.97,1.18) | 1.23 (1.07,1.42) | 0.65 (0.55,0.780 | 0.53 (0.44,0.63) | 1.07 (0.91,1.26) |
| **Other South Asian**  *8,495 (1.1)* | 1.12 (1.07,1.18) | 1.35 (1.18,1.55) | 2,200 (1.1) | 0.94 (0.91,0.98) | 1.08 (0.99,1.17) | 0.55 (0.49,0.62) | 0.57 (0.49,0.66) | 1.03 (0.91,1.18) |
| **Caribbean**  *5,550 (0.7)* | 1.02 (0.95,1.09) | 1.26 (1.10,1.44) | 1,570 (0.8) | 0.99 (0.94,1.05) | 0.94 (0.85,1.04) | 0.63 (0.55,0.71) | 0.45 (0.38,0.52) | 0.89 (0.76,1.04) |
| **African**  *11,379 (1.5)* | 1.02 (0.97,1.07) | 1.49 (1.35,1.65) | 3,118 (1.5) | 0.87 (0.83,0.91) | 0.94 (0.86,1.03) | 0.43 (0.39,0.47) | 0.42 (0.37,0.47) | 0.87 (0.78,0.97) |
| **Other Black**  *3,395 (0.4)* | 0.95 (0.89,1.02) | 1.27 (1.07,1.51) | 812 (0.4) | 0.91 (0.85,0.97) | 0.89 (0.77,1.03) | 0.59 (0.52,0.67) | 0.51 (0.40,0.64) | 0.84 (0.72,0.98) |

**Figure S1. Derivation of study cohort.**

**Clinical Practice Research Datalink**

**N =** **16,990,847**

**BP ≥ 6 months after registration / practice up to standard date & in study period**

*N= 2,831,085*

**BP ≥ 6 months after registration / practice up to standard date & in study period**

*N = 2,765,989*

**No missing covariate data (for smoking or BMI)**

*N = 747,184*

**No missing covariate data (for smoking or BMI)**

*N = 782,141*

**Antihypertensive naïve at 1^st^ raised BP**

*N = 899,901*

**Antihypertensive naïve at 1^st^ raised BP**

*N: 882,859*

**≥ 1 x BP over morbidity-dependent thresholds**

*N = 1,581,890*

**≥ 1 x BP over morbidity-dependent thresholds**

*N = 1,487,239*

**≥ 18 years old at BP**

*N = 2,750,151*

**≥ 18 years old at BP**

*N = 2,715,512*

**≥ 18 years old at study end date (30^th^ June 2019)**

*N = 10,632,759*

**≥ 18 years old at study end date (30^th^ June 2019)**

*N = 4,691,274*

**Missing ethnicity**

*N =* *11,424,727*

**Codes for European, South Asian or African/ African Caribbean ethnicity**

*N = 5,566,120*

**Other ethnicity**

*N=278,979*

**Figure S2. Direct acyclic graph of associations between ethnicity and blood pressure control.**

**
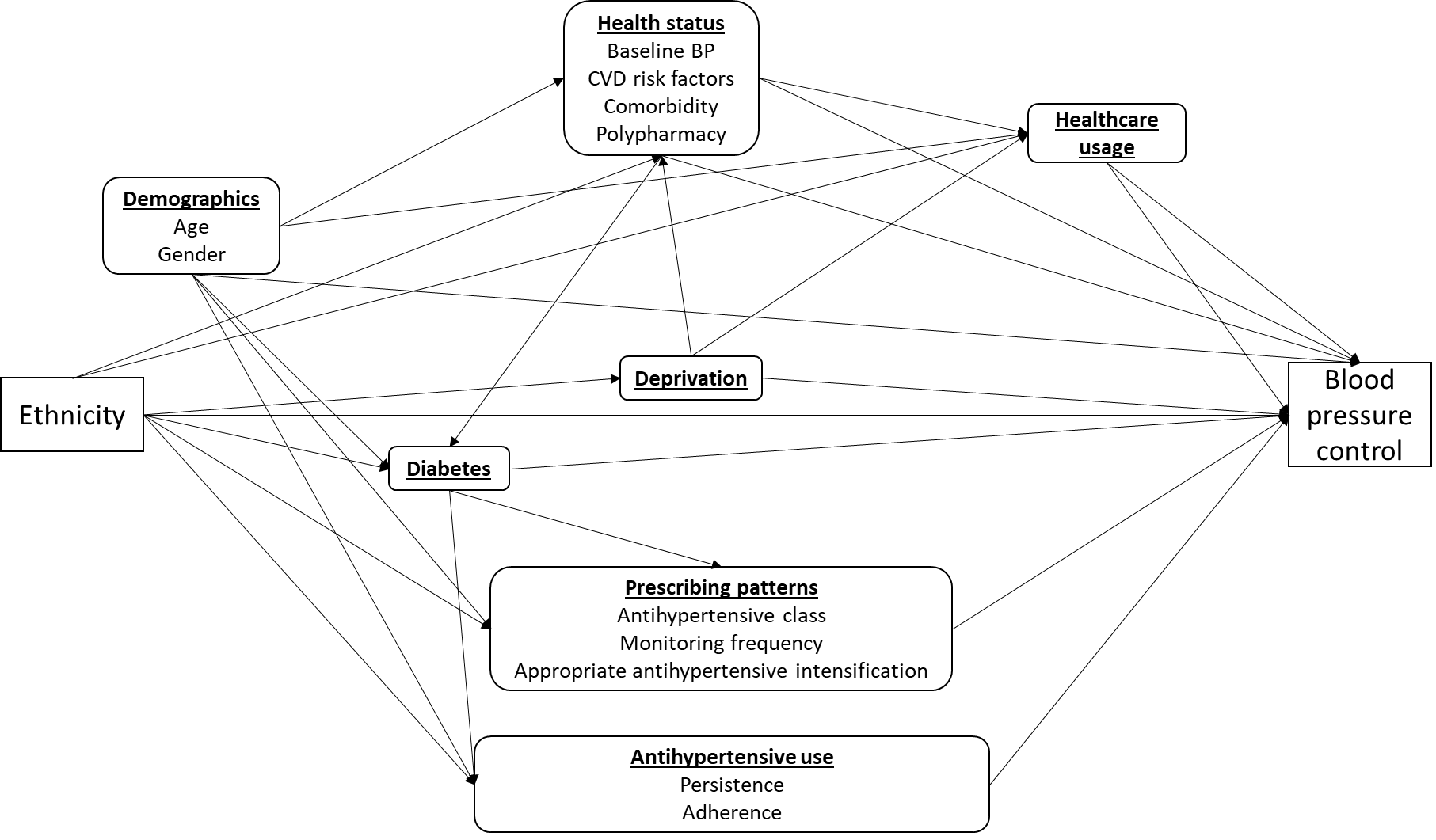
**

**Figure S3. Kaplan-Meier cumulative survival curve for antihypertensive intensification in the first year of antihypertensive treatment, by ethnicity.**

102,322

108,781

116,645

127,939

96,624

161,817

**Figure S4. Sub-group analyses by diabetes status of ethnic differences in hypertension management, antihypertensive usage and BP control at one year; comparisons of people of a) South Asian and b) African/ African Caribbean and European ethnicity.** Data are OR, IRR (BP monitoring rate), or HR (intensification) (95% CI), complete case analysis. Orange capped markers represent estimates for people of South Asian versus European ethnicity, and blue ones for people of African/ African Caribbean versus European ethnicity. Models (fully) adjusted for: age, gender, SBP, deprivation, cardiovascular disease risk factors, comorbidity, healthcare usage and polypharmacy. Persistence and adherence models also adjusted for antihypertensive class, BP control model also adjusted for antihypertensive class, monitoring frequency, intensification and persistence. Intensification data for nested cohort of individuals with a BP exceeding treatment target ≥ 30 days after antihypertensive initiation (N = 161,817). Adherence data for nested cohort of individuals with usable adherence measures (N = 167,620). BP control data for nested cohort with follow-up BP available at 12±6 months after antihypertensive initiation (N = 157,143).

**a)**

**b)**

**Figure S5.** **Differences in hypertension management, antihypertensive usage and BP control at one year by calendar time period.** Data are OR, IRR (BP monitoring rate), or HR (intensification) (95% CI), complete case analysis. Models (fully) adjusted for: age, gender, SBP, deprivation, cardiovascular disease risk factors, comorbidity, healthcare usage and polypharmacy. Persistence and adherence models also adjusted for antihypertensive class, BP control model also adjusted for antihypertensive class, monitoring frequency, intensification and persistence. Intensification data for nested cohort of individuals with a BP exceeding treatment target ≥ 30 days after antihypertensive initiation (N = 161,817). Adherence data for nested cohort of individuals with usable adherence measures (N = 167,620). BP control data for nested cohort with follow-up BP available at 12±6 months after antihypertensive initiation (N = 157,143).

**Figure S6.** **Ethnic differences in hypertension management, antihypertensive usage and BP control at one year by calendar time period.** Data are OR, IRR (BP monitoring rate), or HR (intensification) (95% CI), complete case analysis. Models (fully) adjusted for: age, gender, SBP, deprivation, cardiovascular disease risk factors, comorbidity, healthcare usage and polypharmacy. Persistence and adherence models also adjusted for antihypertensive class, BP control model also adjusted for antihypertensive class, monitoring frequency, intensification and persistence. Intensification data for nested cohort of individuals with a BP exceeding treatment target ≥ 30 days after antihypertensive initiation (N = 161,817). Adherence data for nested cohort of individuals with usable adherence measures (N = 167,620). BP control data for nested cohort with follow-up BP available at 12±6 months after antihypertensive initiation (N = 157,143).

**Supplementary material: references**

1. Williams B, Poulter NR, Brown MJ, et al. Guidelines for management of hypertension: report of the fourth working party of the British Hypertension Society, 2004-BHS IV. *J Hum Hypertens* 2004; **18**(3): 139-85.

2. Sever P. New hypertension guidelines from the National Institute for Health and Clinical Excellence and the British Hypertension Society. *J Renin Angiotensin Aldosterone Syst* 2006; **7**(2): 61-3.

3. Bhopal R. Glossary of terms relating to ethnicity and race: for reflection and debate. *J Epidemiol Community Health* 2004; **58**(6): 441-5.

4. Andrade SE, Kahler KH, Frech F, Chan KA. Methods for evaluation of medication adherence and persistence using automated databases. *Pharmacoepidemiol Drug Saf* 2006; **15**(8): 565-74; discussion 75-7.

5. Ministry of Housing CaLG. English indices of deprivation. 2015. <https://www.gov.uk/government/statistics/english-indices-of-deprivation-2015> (accessed 27th January 2022).

6. Eastwood SV, Mathur R, Atkinson M, et al. Algorithms for the Capture and Adjudication of Prevalent and Incident Diabetes in UK Biobank. *PLoS One* 2016; **11**(9): e0162388.

7. Farmer R, Mathur R, Bhaskaran K, Eastwood SV, Chaturvedi N, Smeeth L. Promises and pitfalls of electronic health record analysis. *Diabetologia* 2018; **61**(6): 1241-8.

8. White IR, Carlin JB. Bias and efficiency of multiple imputation compared with complete-case analysis for missing covariate values. *Stat Med* 2010; **29**(28): 2920-31.
